# The coupling and competition of crystallization and phase separation, correlating thermodynamics and kinetics in OPV morphology and performances

**DOI:** 10.1038/s41467-020-20515-3

**Published:** 2021-01-12

**Authors:** Zaiyu Wang, Ke Gao, Yuanyuan Kan, Ming Zhang, Chaoqun Qiu, Lei Zhu, Zhe Zhao, Xiaobin Peng, Wei Feng, Zhiyuan Qian, Xiaodan Gu, Alex K.-Y. Jen, Ben Zhong Tang, Yong Cao, Yongming Zhang, Feng Liu

**Affiliations:** 1 Frontiers Science Center for Transformative Molecules, In-situ Center for Physical Science, and Center of Hydrogen Science, School of Chemistry and Chemical Engineering, Shanghai Jiao Tong University, 200240 Shanghai, China; 2 Department of Chemistry, The Hong Kong University of Science and Technology, Clear Water Bay, 999077 Kowloon, Hong Kong China; 3 Department of Materials Science and Engineering, University of Washington, Seattle, WA 98195 USA; 4 State Key Lab of Luminescent Materials and Devices, South China University of Technology, 510640 Guangzhou, China; 5 State Key Laboratory of Fluorinated Functional Membrane Materials and Dongyue Future Hydrogen Energy Materials Company, 256401 Zibo, Shandong China; 6 School of Polymer Science and Engineering, Center for Optoelectronic Materials and Devices, The University of Southern Mississippi, Hattiesburg, MS 39406 USA; 7 Department of Chemistry, City University of Hong Kong, 999077 Kowloon, Hong Kong China

**Keywords:** Organic molecules in materials science, Surfaces, interfaces and thin films, Solar cells, Organic molecules in materials science

## Abstract

The active layer morphology transition of organic photovoltaics under non-equilibrium conditions are of vital importance in determining the device power conversion efficiency and stability; however, a general and unified picture on this issue has not been well addressed. Using combined in situ and ex situ morphology characterizations, morphological parameters relating to kinetics and thermodynamics of morphology evolution are extracted and studied in model systems under thermal annealing. The coupling and competition of crystallization and demixing are found to be critical in morphology evolution, phase purification and interfacial orientation. A unified model summarizing different phase diagrams and all possible kinetic routes is proposed. The current observations address the fundamental issues underlying the formation of the complex multi-length scale morphology in bulk heterojunction blends and provide useful morphology optimization guidelines for processing devices with higher efficiency and stability.

## Abstract (web summary)

Designing efficient blue perovskite LEDs by using mixed halides perovskite is still a challenge, limited mainly by the phase segregation issue. Here, the authors demonstrate in situ fabrication of quasi-2D CsPbClBr2 nanocrystal films with mixed ligands to overcome the constraint.

## Introduction

Thin-film organic photovoltaics (OPVs) are transformative and ecologically low footprint alternatives to conventional photovoltaic technologies, whose commercialization depends on breakthroughs in understanding the structure–property relationship and developing scalable synthesis and processing strategies that are robust, environmentally friendly, economically viable, and sustainable^1–4^. Various novel OPV materials have been developed so far to meet the needs of absorbing different regions of the solar spectrum and ensuring donor/acceptor energy level alignment to achieve high-power conversion efficiency (PCE)^5–13^. In OPV devices, the morphology of the active layer, comprising blends of polymer/oligomer/small-molecule donors and acceptors plays a critical role. The ideal OPV active layer is a bulk heterojunction (BHJ) bicontinuous-interpenetrating network formed by donor and acceptor segregation, which affords efficient charge generation and transport simultaneously^14,15^. The processing conditions including the properties of the solvents and additives, the rate of solvent evaporation, and annealing treatments are common factors that affect the active layer morphology^16–22^. In real cases, a delicate balance between multiple kinetic processes and thermodynamics factors defines the final morphology, yielding a multi-length scale phase separation, giving rise to improved performance. Therefore, molecular orientation, crystallinity, interfacial orientation, and phase separation need to be studied in order to get a robust structure-property relationship^23–25^.

BHJ thin-film morphology can be complex^26,27^. Small changes in materials’ chemical structure and processing condition can lead to dramatic difference in morphology and device efficiency^22,28,29^. Thermodynamic equilibrium state can hardly be achieved under fast spin casting or mild post-annealing process, whereas kinetic phase transition pathways play a significant role in determining the final morphology^30,31^. The solution-cast OPV films usually adopt a kinetically trapped morphology due to rapid isothermal quenching solidification process upon the fast removal of solvents^32,33^. Even in the simplest binary donor/acceptor mixture, the solidification process from solvent evaporation can hardly be described based on interactions within a binary blend only, because solvent interaction with solutes need to be evaluated, which requires the use of a ternary phase diagram to address the issue. Competing solidification mechanisms, such as precipitation, vitrification, crystallization, demixing etc. need to be coupled with solubility and free energy landscape to shape out the multi-length scale morphology. Notwithstanding the success of morphology control to improve solar cell PCE, fundamental issues regarding the thermodynamics and kinetics of morphology transition in BHJ thin films are barely addressed. It is important to study the common physical chemistry of BHJ phase separation that can be applied to understand the morphology evolution process. Such effort would require detailed comparisons across multiple systems that are simple and can be readily analyzed.

In this work, we devote efforts to investigate morphology transition mechanisms of spin-cast BHJ thin films. Post thermal annealing is used to induce morphology change, which decouples the difficulty brought by solvent evaporation. We use this simple platform to investigate the structure evolution since the kinetic processes and thermodynamic attributes can be readily monitored in a controllable manner. Small-molecule donor and fullerene acceptor binary blends are chosen as the model systems to simplify the study due to well-defined physical properties and easier probing capabilities, and the results can be widely applied due to generality in consideration and observation. In situ and ex situ grazing-incidence small- and wide-angle X-ray scattering (GISAXS/GIWAXS) characterizations are carried out to study the kinetics and thermodynamics of phase separation and crystallization^19,33–35^. We note that the crystallization and demixing are coupled and in competition, determined by the glass transition and miscibility gap of BHJ thin film. Crystallization and diffusion of the donor molecules in a moderate temperature region determine the length scale of phase separation, phase purity, and interfacial orientation, which are decisive factors that control short-circuit current (*J*_SC_) and fill factor (FF) of solar cells. Prolonged and high-temperature annealing is detrimental to device performance due to more rigorous phase separation that is far from ideal. The morphology details show good correlation with device performance parameters, yielding solid conclusions that shape the morphology-performance relationship. Extended efforts are applied to most recent high-performance non-fullerene acceptor (NFA)-based BHJ solar cells, forming unified conclusions. Such results of detailed morphology features together with the insights of phase separation thermodynamics and kinetics can be used as a general guideline in understanding the morphology and optimization of OPV devices.

## Results

**Fig. 1:**
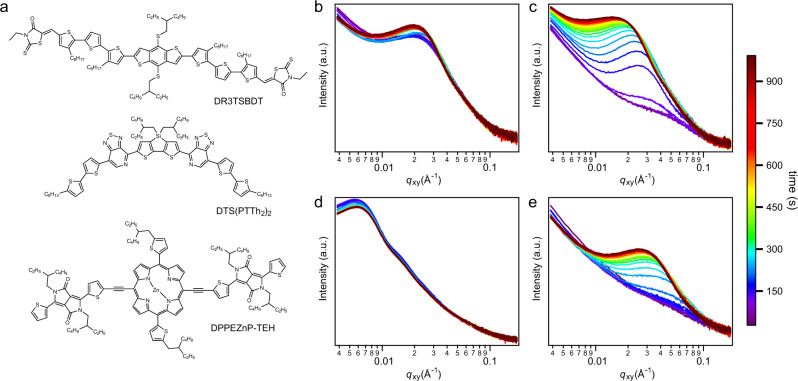
Chemical structure and in situ grazing-incidence small-angle X-ray scattering (GISAXS). **a** Chemical structure of DR3TSBDT, DTS(PTTh_2_)_2_ and DPPEZnP-TEH. **b**–**e** In situ GISAXS in-plane profiles of **b** DR3TSBDT:PC_71_BM, **c** DTS(PTTh_2_)_2_:PC_71_BM, **d** DPPEZnP-TEH:PC_61_BM and **e** DPPEZnP-TEH:PC_61_BM/Py (scattering data was collected every 30 s with 2 s exposure time).

We choose several well-known OPV blends, viz. DR3TSBDT:PC_71_BM, DTS(PTTh_2_)_2_:PC_71_BM and DPPEZnP-TEH:PC_61_BM (molecular structures shown in Fig. 1a), which show different crystallization and phase separation behaviors under different temperature backgrounds. DR3TSBDT:PC_71_BM blend performs well both in as-cast and thermal annealing conditions, and the simple linear thiophene-based backbone results in high-crystalline characteristic in DR3TSBDT^36^. DTS(PTTh_2_)_2_:PC_71_BM blend shows good performance under thermal annealing and additive processing, whereas the device barely works in as-cast condition^37,38^. DPPEZnP-TEH:PC_61_BM blend shows extensive phase separation upon casting from chlorobenzene (CB) solution, but the system mixes quite well when 1% pyridine (Py) additive is used^20,21^. The current four materials groups cover varied morphological features in as-cast films, which can be used to study the phase behavior under thermal annealing to explore thermodynamic and kinetic influences. Detailed device comparison has been carried out together with morphological investigation, which will be discussed in detail.

### In situ X-ray scattering characterization

To observe the morphology evolution upon annealing in real time, a heating plate with precise temperature control was integrated with GISAXS facility to study the lateral phase separation. Experiments were carried out in helium atmosphere to avoid sample degradation and air scattering. In experiments, samples were heated from 40 °C at a rate of 20 °C min^−1^; and then maintained at the target temperature (100 or 110 °C). Meanwhile, scattering data was collected every 30 s with a 2 s exposure time for one frame (profiles exhibited in Fig. 1b–e). From in situ GISAXS results, we see that the scattering profiles of DR3TSBDT:PC_71_BM (Fig. 1b) and DPPEZnP-TEH:PC_61_BM (Fig. 1d) remain almost unchanged during thermal annealing, while DTS(PTTh_2_)_2_:PC_71_BM (Fig. 1c) and DPPEZnP-TEH:PC_61_BM using pyridine additive (Fig. 1e) exhibit progressive phase separation as indicated by the continuous low-*q* shifting of the scattering peak. Heating temperature, peak intensity and phase separation distance as a function of time are summarized in Supplementary Fig. 1. Initially, DR3TSBDT:PC_71_BM blend film shows a well-defined peak located at *q* ≈ 0.02 Å^−1^ (*T* = 40 °C), corresponding to a phase separation distance of ~32 nm. Such phase separation size and intensity do not change much under subsequent heating (from 40 to 100 °C) and annealing. For DTS(PTTh_2_)_2_:PC_71_BM blend film, there is no discernible peak observed in the measured *q*-range during heating from 40 to 70 °C. Moreover when heated to 80 °C, a weak shoulder at *q* ≈ 0.026 Å^−1^ (corresponding to a phase separation distance of ~24 nm) arises and then shifts to lower *q*, and becomes more evident with increased intensity. When the temperature gets stabilized at 110 °C, the peak position continues to move towards low *q* with enhanced intensity yet at reduced speed. For as-cast DPPEZnP-TEH:PC_61_BM blend film, the mixture has already formed large phase separation with a peak at *q* ≈ 0.0056 Å^−1^ (corresponding to a phase separation distance of ~112 nm). For DPPEZnP-TEH:PC_61_BM/Py film, no phase separation peak is seen before heating temperature reaches 100 °C. Afterwards, a weak shoulder turns up and then develops into a well resolved peak. In situ GISAXS results categorize the four systems into two groups: (1) DR3TSBDT:PC_71_BM and DPPEZnP-TEH:PC_61_BM blend films that have well-developed phase-separated morphology, and thermal annealing does not strongly affect their phase separation; (2) DTS(PTTh_2_)_2_:PC_71_BM and DPPEZnP-TEH:PC_61_BM/Py blends are well-mixed upon casting and thermal annealing induces the phase separation.

**Fig. 2:**
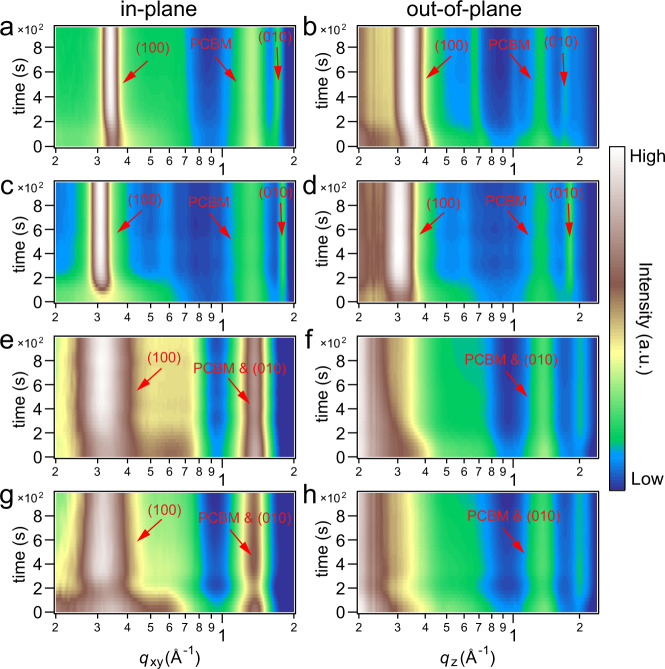
In situ grazing-incidence wide-angle X-ray scattering (GIWAXS). In situ GIWAXS in-plane and out-of-plane pseudo color mapping plots of **a**, **b** DR3TSBDT:PC_71_BM, **c**, **d** DTS(PTTh_2_)_2_:PC_71_BM, **e**, **f** DPPEZnP-TEH:PC_61_BM and **g**, **h** DPPEZnP-TEH:PC_61_BM/Py (scattering data was collected every 30 s with 2 s exposure time).

In situ GIWAXS was used to investigate the materials’ crystallization under thermal annealing. Results of in-plane (IP) and out-of-plane (OOP) color-mapping plots are shown in Fig. 2 (corresponding line profiles shown in Supplementary Fig. 2). DR3TSBDT:PC_71_BM blend film (Fig. 2a, b) is highly crystalline in as cast thin film, which shows well-defined (100) peak as well as weak (010) peak in IP and OOP direction. The rising temperature enlarges the (100) packing distance, as seen by the shifting of (100) peak profiles, and the peak shape becomes sharper. (010) peak under thermal annealing shifts slightly to higher *q* position, creating better pathways for carrier transport with more compact packing. The PC_71_BM peak centered at *q* ≈ 1.34 Å^−1^ remains similar in its shape and position, with isotropic feature during annealing. This is because the annealing temperature is below the glass transition temperature (*T*_g_) of PC_71_BM, and cold crystallization of PC_71_BM is neglegible^34,39,40^. Crystallization behavior of DTS(PTTh_2_)_2_:PC_71_BM mixture is different (shown in Fig. 2c, d). The OOP (100) and IP (010) reflections are quite weak when heated from 40 to 70 °C. When heated to 80 °C, these peaks become obvious. Afterwards, their development becomes similar to that of DR3TSBDT:PC_71_BM. The crystallinity of DPPEZnP-TEH is moderate, and thus its (100) reflection is weak even in pure film^20^. Besides, π-π stacking distance of DPPEZnP-TEH is larger than that of common conjugated molecules, with the (010) diffraction peak centered at *q* ≈ 1.48 Å^−1^ close to PC_61_BM peak, which makes it difficult to analyze. In large phase-separated DPPEZnP-TEH:PC_61_BM film, there is little structure change upon thermal annealing. For DPPEZnP-TEH:PC_61_BM/Py film, the IP (100) peak becomes pronounced gradually, whereas the OOP profiles remain almost unchanged. Scherrer equation is used to extract the crystal coherence length (CCL) or crystal size from GIWAXS profiles (shown in Supplementary Fig. 3)^41–43^. Besides, paracrystallinity *g*-factor (shown in Supplementary Fig. 4) representing accumulative structural disorder was also calculated^44,45^. They will be discussed below together with other morphology parameters.

**Fig. 3:**
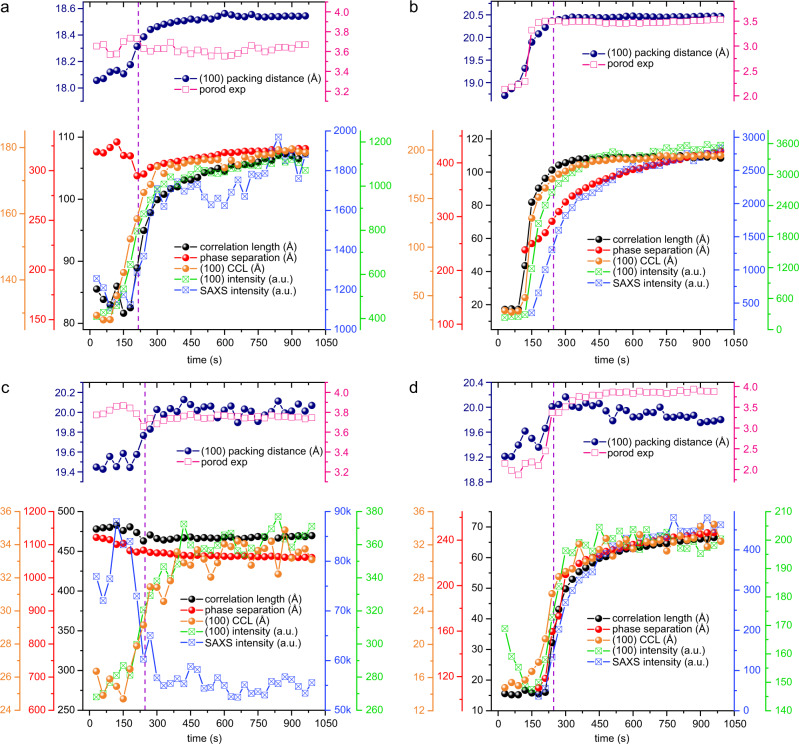
Fitting results of morphology parameters extracted from in situ GISAXS/GIWAXS profiles. **a** DR3TSBDT:PC_71_BM, **b** DTS(PTTh_2_)_2_:PC_71_BM, **c** DPPEZnP-TEH:PC_61_BM, and **d** DPPEZnP-TEH:PC_61_BM/Py (purple dashed line is used to separating heating stage from annealing stage).

As previously mentioned, the morphology of BHJ blend film is of non-equilibrium nature and multiple thermodynamic and kinetic factors dictate the final morphology. The coupling and competition between different phase transition mechanisms could lead to nanostructure of multiple length scales, bring in the richness of BHJ morphology. Therefore, we analyze the in situ scattering data in a quantitative manner through fitting to extract detailed structural information, trying to decouple the crystallization and phase separation processes. These related parameters are summarized in Fig. 3, in which we use (100) packing distance, (100) CCL and (100) intensity to monitor the crystallization process, and phase separation peak distance and SAXS intensity to track phase separation change. DR3TSBDT:PC_71_BM blend is of high-crystalline nature, which shows pronounced crystalline diffraction peak in as-cast thin film. Although thermal annealing does not change the global phase separation, the crystalline ordering is increased. The (100) packing distance starts to increase at 40 °C, which then rises quickly at 80 °C. Such a structural reorganization indicates that thin film has passed through the glass transition, and thus molecular mobility increases to induce cold crystallization. The (100) CCL and scattering intensity develop simultaneously, indicating that the new crystallization occurred via the packing of molecules onto the growth front of existing crystallites, following the typical crystal growth mechanism as opposed to the nucleation of new crystallites^46^. The *g*-factor slightly decreases from 14.2% to 12.2%, indicating that cumulative disorder is alleviated. The length scale of phase separation remains quite steady, with only a slight reduction in quick crystal growth transition period (from 40 to 80 °C), indicating that the morphology framework does not change. The correlation length, which marks the typical size scale of the mesh network structure, develops in the same trend with crystal growth, revealing that crystalline domain constitutes one phase in morphology. Thus, morphology evolution in DR3TSBDT:PC_71_BM blend during thermal annealing is a later stage of crystallization-induced phase purification process, in which the donor crystal growth depletes the PC_71_BM and cause donor concentration impoverishment in the remaining mixed region. Such a growth process does not create high-interfacial coarsening as we see a quite steady Porod exponent (~3.6) is obtained. Thus, the diffusion of donor molecule towards growth front and the subsequent crystallization are efficient. Such a purification process pushes PC_71_BM to get enriched at the interface between the donor crystallites and the mixed region, which elevates the *T*_g_ of the interface and also the mixed region. Finally, the system is jammed by the donor deficient vitrified mixed region that frustrates the further donor diffusion and crystallization^47^.

The as-cast DTS(PTTh_2_)_2_:PC_71_BM blend film has no observable phase separation and the initial crystallinity is low. Shown in Fig. 3b, the (100) packing distance, intensity, CCL, Porod exponent, and correlation length remain unchanged or grow slowly when heated from 40 to 80 °C. BHJ thin film is kept at a good mixing state and cumulative disorder is very large as reflected by the large *g*-factor of 27.8%. This indicates that the temperature region is still in the vitrification zone (below *T*_g_) where molecular motion is largely prohibited. Sharp turn-ups in all these parameters are observed when temperature goes over 80 °C and slow down when getting to 90 °C. The concurrence of (100) intensity, CCL and correlation length increase, and *g*-factor dramatic drop indicates that crystallization dominates the structure evolution. The size and intensity of phase separation lag slightly behind the crystallization process, indicating it is a diffusion-limited process that transport of donor molecule slows down when the mixed region gets depleted and thus the definition of density fluctuation goes slower than crystallization. Porod exponent develops in pace with crystallization, from mass fractal region to surface fractal region when crystallites present and then get stabilized. Therefore, DTS(PTTh_2_)_2_:PC_71_BM blend and DR3TSBDT:PC_71_BM blend share quite similar mechanism of crystallization-induced phase separation, except that the initial states of as-cast thin film are different.

The DPPEZnP-TEH:PC_61_BM blend film without pyridine (Fig. 3c) shows large phase separation of ~112 nm prior to heating. During thermal annealing, the phase separation and correlation length remain stable except for slight reduction at the heating stage. The CCL is far below the correlation length and size of phase separation. This mismatch indicates that phase transition is dictated by factors other than crystallization. The (100) packing distance and CCL first increase upon heating and gradually become stable, with an enlargement around 3% and 26%, respectively. Such change happens within the phase-separated morphology and thus cannot induce the global morphology change. Porod exponent remains stable around 3.8, indicating a quite sharp interface of phase-separated structure. The DPPEZnP-TEH:PC_61_BM blend film with 1% pyridine additive shows a well-mixed single phase morphology and weak crystallinity at the initial state. As shown in Fig. 3d, the (100) CCL exhibits quick increase during 80–110 °C region, with approximate 110% increase from 15 to 32 Å during annealing. Before temperature rises to 110 °C, the correlation length remains unchanged, and both (100) packing distance and Porod exponent show very slow increase below 80 °C, which then quickly jumps from 80 °C to 110 °C. Such process goes along with GISAXS intensity. The CCL at low temperature (below 80 °C) is similar to correlation length; while in high-temperature region, it is much smaller than correlation length and the length scale of phase separation. Such difference indicates that crystallization is the major driving force in morphology change in early stage; and in later stage, global phase separation occurs and surpass the crystal-induced phase separation. The latter process yields a quite sharp Porod exponent of ~3.8, and thus such phase separation induces a sharp interface. Therefore, DPPEZnP-TEH:PC_61_BM/Py blend film undergoes coupling and competing phase separation mechanism of crystallization and demixing.

### Thermodynamic miscibility of donor/acceptor blend

While in situ experiments reveal the important kinetics of crystallization and phase separation, the influence of thermodynamic factors on these processes should also be considered. The thermodynamic mixing behavior of small molecule and fullerene in a binary mixture can be analyzed using the mean field theory statistics developed by Flory et al to estimate the interaction parameter^48,49^. By analyzing melting point depression in crystalline materials when blended with impurities due to the reduction of chemical potential, the Flory–Huggins interaction parameter can be extracted following Eq. (1) (details in Supplementary Note 5)^50,51^:

1 $$\frac{1}{{T_{\mathrm{m}}}} - \frac{1}{{T_{\mathrm{m}}^0}} =	 - \frac{{RV_{2{\mathrm{u}}}}}{{\Delta H_{2{\mathrm{u}}}V_{1{\mathrm{u}}}}}\Bigg[\frac{{\ln \nu _2}}{{m_2}} + \left(\frac{1}{{m_2}} - \frac{1}{{m_1}} \right) \times ({1 - \nu_2}) \\ 	+ \chi_{12}({1-\nu_2})^2\Bigg],$$

where $T_m^0$ represents the melting point of host matter in the standard state, *T*_m_ is the melting point when mixed with impurities, *R* is the gas constant, the subscript 1 identified with the impurities and 2 with the host matter, *V*_u_ is the molar volume (of repeating unit for polymer), Δ*H*_u_ is enthalpy of fusion per mole (of repeating unit for polymer), *v* is the volume fraction, *m* is the degree of polymerization, and *χ*_12_ represents the host-impurities interaction parameter. Besides, Hansen solubility parameter method is also used to estimate the *χ* value that only takes account of material chemical structure^40^.

These methods yield two sets of *χ* results (tabulated in Table 1) that describe the miscibility between donor and acceptors based on different theoretical backgrounds. The relative small *χ* of DR3TSBDT:PC_71_BM and DTS(PTTh_2_)_2_:PC_71_BM suggests that they have good miscibility with PC_71_BM and are prone to mix with PC_71_BM. DPPEZnP-TEH:PC_61_BM blend has a larger *χ*, which leads to strong phase separation in as-cast thin film. The presence of pyridine additive could change the interaction between materials and form a good mixture. The subsequent thermal heating above *T*_g_ could quickly drive the blend to undergo strong demixing (most possibly through spinodal decomposition (SD)) beyond crystallization-induced morphology change. The current results show that the thermodynamic interaction parameter *χ* can only conditionally correlate with phase separation in as-cast thin film. This is because during BHJ film formation from solution casting, the quick evaporation of solvent quickly precipitates out solutes, forming kinetically trapped morphologies. Therefore, it is inadequate to use only interaction parameter *χ* to predict the phase behavior of BHJ thin film with strong crystallinity; however, the interaction parameter *χ* is valuable to the thermal annealing treatments, since it can qualitatively estimate miscibility at a certain temperature and point out the mixing/demixing tendency, and is useful in studying amorphous systems^52^.

**Table 1:** Materials interaction parameters (*χ*) obtained by different methods and phase separation length (*L*) at different processing conditions.

	*χ*_MDP_^a^	*χ*_cal_^b^	As-cast morphology	*L*_as-cast_ (nm)	*L*_100 °C_ (nm)	*L*_180 °C_ (nm)
DR3TSBDT: PC_71_BM	0.285	1.17	Phase separated	32	32	220
DTS(PTTh_2_)_2_: PC_71_BM	1.157	2.37	Mixed	N/A	44	350
DPPEZnP-TEH:PC_61_BM	N/A	6.16	Phase separated	112	110	108
			Mixed^c^	N/A^c^	24^c^	40^c^

^a^*χ* obtained from melting point depression.

^b^*χ* calculated from Hansen solubility parameters.

^c^DPPEZnP-TEH:PC_61_BM with pyridine.

### Ex situ X-ray scattering under different annealing temperature

**Fig. 4:**
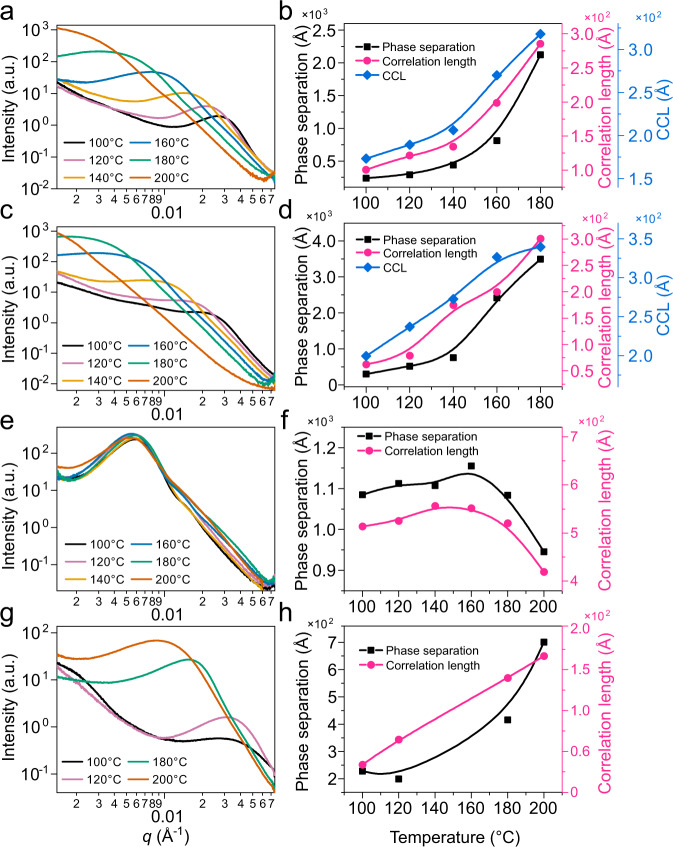
Ex situ resonant soft X-ray scattering (RSoXS) and relevant morphology parameters. RSoXS profiles and morphology parameters summary of **a**, **b** DR3TSBDT:PC_71_BM, **c**, **d** DTS(PTTh_2_)_2_:PC_71_BM, **e**, **f** DPPEZnP-TEH:PC_61_BM and **g**, **h** DPPEZnP-TEH:PC_61_BM/Py under different annealing temperature.

Considering the relative small *χ* value, in DR3TSBDT:PC_71_BM and DTS(PTTh_2_)_2_:PC_71_BM blends, it is typical crystallization-induced phase separation^46^, which can be a situation that the system goes into the metastable region in phase diagram and initiate nucleation and growth (NG, also called binodal decomposition (BD)), or simply the blends go over the glass transition line to induce cold crystallization^53^. We then carry out ex situ annealing experiments under elevated temperatures to reveal the temperature dependence of the morphology. BHJ thin films were annealed at each temperature for 10 min to stabilize the morphology and then measure the phase separation using resonant soft X-ray scattering (RSoXS), which can provide large accessible *q* range using low-energy photon (284.2 eV). Complementary GIWAXS experiments are carried out on DR3TSBDT:PC_71_BM and DTS(PTTh_2_)_2_:PC_71_BM blends to investigate molecular ordering due to their strong crystallization tendency. As seen in Fig. 4 and Supplementary Fig. 10, the length scale of phase separation for BHJ thin films change progressively towards low *q* region at higher temperature, except DPPEZnP-TEH:PC_61_BM blend processed without additive. When thermal annealing at 200 °C that is far above the thin film *T*_g_, the nearly fixed phase separation indicates that the system is locked by mechanism other than SD, which is most probably due to surface pinning effects^54,55^. In GIWAXS characterization, DR3TSBDT in blend thin film shows diffraction spots when annealed over 140 °C, which get intensified if temperature goes further up. The crystal size shows quicker turn-up at 140 °C. DTS(PTTh_2_)_2_ also shows a crystal orientation change when annealed above 140 °C. Thus, molecules are easier to order and adopt preferential orientation since molecular mobility is largely enhanced. The length scale of phase separation and temperature correlation is investigated, from which a size changing turning point is seen at 140 °C, which is close to the *T*_g_ of PCBM^39,40^. The quickly enlarged phase separation that goes well above the crystal size in BHJ blends in high-temperature annealing indicates that demixing other than crystallization dictates morphology, which could most possibly be SD. Small crystals migrate and agglomerate to form larger domains, which constitutes the scatters in a PCBM rich matrix. The quick change in phase separation size scales above 140 °C indicates a mechanism change in phase separation. The glass transition of donor materials is usually low, and in blends should be well below that of PCBM *T*_g_. While crystallization-induced phase separation is well recorded in in situ experiments, the temperature of phase separation slope change marks a transition from crystallization-induced phase separation to spinodal decomposition phase separation. Therefore, DR3TSBDT:PC_71_BM and DTS(PTTh_2_)_2_:PC_71_BM most likely take a lower critical solution temperature (LCST) phase diagram. Similar morphology change is seen in DPPEZnP-TEH:PC_71_BM blend in order to exclude the effect of PCBM change. As shown in Supplementary Fig. 8, there is no phase separation until the annealing temperature reaches 100 °C, and the phase separation distance increases quickly after 160 °C. The CCL increases from 40 to 120 °C with a quick turn-up at 120 °C. The slightly higher temperature transition in DPPEZnP-TEH:PC_71_BM blend is due to the slightly higher *T*_g_ for PC_71_BM.

### Discourse of phase separation mechanism

**Fig. 5:**
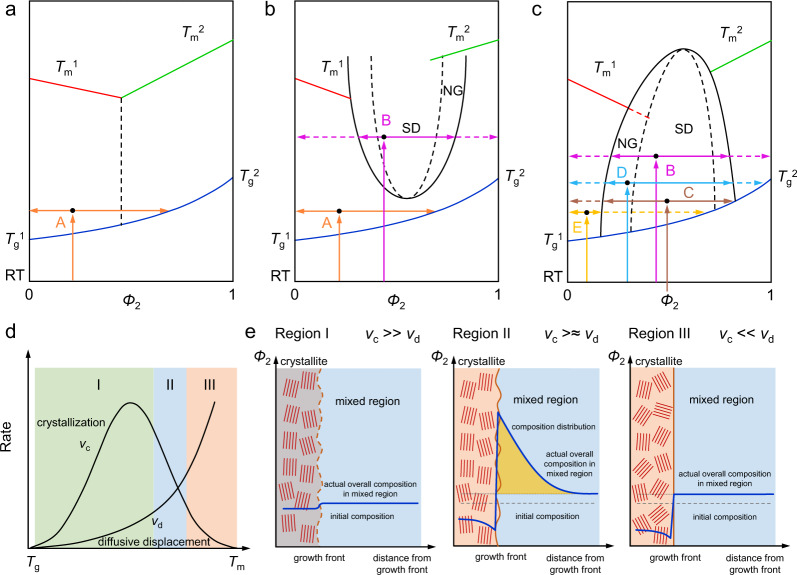
Schematic thermodynamics and kinetics pathways under different thermal stress. **a**–**c** Schematic non-equilibrium phase diagrams of two crystallizable components with **a** pseudo-eutectic feature, **b** lower critical solution temperature (LCST) feature, and **c** upper critical solution temperature (UCST) feature. The capitals A to E and corresponding colored line with arrows represent five possible phase transition routes. Melting point line are denoted by red line (*T*_m_^1^) and green line (*T*_m_^2^). Glass transition line is indicated by curves in navy blue. Miscibility gap is surrounded by black solid curve. Inside miscibility gap, black dashed line is used to separate spinodal decomposition (SD) region from nucleation and growth (NG) (also called binodal decomposition (BD)) region. **d** Schematic sketch of crystallization (*v*_c_) and diffusive displacement (*v*_d_) dependence on temperature between glass transition temperature (*T*_g_), and melting temperature (*T*_m_). **e** Composition profiles at growth front of three regions indicated in **d**. Horizontal axis represents the distance from the growth front (indicated by orange curve). Vertical axis represents the content of component (denoted as *Φ*_2_) with higher *T*_g_ or simply amorphous. Composition distribution is indicated by solid blue curve, whereas initial composition and actual overall composition in mixed region are indicated by dash gray line and dot gray line, respectively.

The above discussed in situ and ex situ annealing experiments reveal the important aspects of phase transition mechanisms in BHJ blends. A unified framework will be constructed to integrate the current experimental data together with previous investigations on OPV blends. Let us begin with the simplest case, the system that is annealed above the *T*_g_ with no miscibility gap. Assuming both donor and acceptor are semi-crystalline (a single crystallizable component with an amorphous component is easier to deal with, and if the second component with higher *T*_g_ does not crystallize at the treatment temperature, we can treat it as an equivalent amorphous part), this system could adopt a non-equilibrium phase diagram with no miscibility gap in the whole range, exhibiting a pseudo-eutectic feature (Fig. 5a) or with a LCST (Fig. 5b). In this condition, *T*_g_ turns out to be an important factor that not only gauges the initial step of phase separation induced by crystallization but also results in the deceleration of the crystallization caused by the vitrification due to the composition dependent *T*_g_^56^. This self-deceleration crystallization with non-linear growth rate deviates from the Avrami behavior since the crystallization rate (*ν*_c_) is not a constant. Indeed, the crystallization kinetics under specific annealing temperature or crystallization temperature (*T*_c_) is a competition between the diffusive displacement rate (*ν*_d_) and the crystallization rate (*ν*_c_), which leads to different composition profiles at the growth front. *ν*_d_ increases with temperature, and its temperature dependence can be treated by Williams–Landel–Ferry (WLF) equation (Eqs. (2) and (3))^57^,

2 $$\upsilon _d\left( {T_{\mathrm{c}}} \right) = \frac{d}{{dt}}\left[ {r^2\left( {T_{\mathrm{c}},t} \right)} \right]^{1/2} = \frac{d}{{dt}}\left[ {6D\left( {T_{\mathrm{c}}} \right)t} \right]^{1/2} = \frac{1}{2}\sqrt {6D\left( {T_{\mathrm{c}}} \right)} \,t^{ - \frac{1}{2}},$$

3 $$D\left( {T_{\mathrm{c}}} \right) \approx D^0{\mathrm{exp}}\left[ {\frac{{c_1\left( {T_{\mathrm{c}} - 50 - T_{\mathrm{g}}} \right)}}{{c_2 + T_{\mathrm{c}} - 50 - T_{\mathrm{g}}}}} \right] = D^0{\mathrm{exp}}\left[ {\frac{{ - \Delta G_d\left( {T_{\mathrm{g}},T_{\mathrm{c}}} \right)}}{{RT_{\mathrm{c}}}}} \right],$$

where *D* (*T*_c_) is the whole chain diffusion constant, <*r*^2^ > is the mean squared displacement of a chain, *c*_1_ and *c*_2_ are constants. The *ν*_c_ is determined by Eq. (4)^58,59^,

4 $$\upsilon _c\left( {T_{\mathrm{c}}} \right) = \upsilon _{\mathrm{c}}^0\phi _{\mathrm{c}}{\mathrm{exp}}\left[ {\frac{{ - k_{\mathrm{c}}}}{{T_{\mathrm{c}}\left( {T_{{\mathrm{m,m}}} - T_{\mathrm{c}}} \right)f}} + \frac{{c_3T_{{\mathrm{m,m}}}\ln \phi _{\mathrm{c}}}}{{\left( {T_{m,m} - T_{\mathrm{c}}} \right)}}} \right] \\ {\mathrm{exp}}\left[ {\frac{{ - \Delta G_d\left( {T_{\mathrm{g}},T_{\mathrm{c}}} \right)}}{{RT_{\mathrm{c}}}}} \right] = F_{\mathrm{p}} \cdot F_{\mathrm{n}} \cdot F_{\mathrm{e}} \cdot F_{\mathrm{v}}$$

where *ϕ*_c_ is the volume fraction of the crystallizing component with lower *T*_g_, *T*_m,m_ is the melting temperature of the blend, *k*_*c*_, *f*, and *c*_3_ are constants. *F*_p_ is a prefactor, and *F*_n_, *F*_e_, and *F*_v_ are nucleation, entropic (i.e., dilution), and vitrification contributions, respectively. The relation of the crystallization rate (*ν*_c_) and diffusive displacement rate (*ν*_d_) is shown in Fig. 5d. Depending on this relationship, the three regions can be defined (Fig. 5e). If *ν*_d_ ≪ *ν*_c_, which is usually occurred when one component in the system crystallize under a low *T*_c_, the system is strongly diffusion limited. The component with higher *T*_g_ will be expelled by the crystallization of the component with lower *T*_g_, and get trapped in the inter-crystalline regions (Region I). The composition profiles of the mixed region beyond the crystallite region will remain the same as the initial composition. Such morphology is expected to be unfavorable for device optimization since the crystallization process is limited that can only form small and isolated crystalline domains, which does not help with hole transport. When rising the *T*_c_ to an intermediate range, the diffusion and crystallization rate increases to a comparable level and keep a *ν*_d_ ≥ *ν*_c_ situation (Region II). Thin film crystallizes much stronger to form an interconnected domain. The high *T*_g_ component is expelled from the crystallization front and will be diffusive composition profile in the mixed region due to the Fickian diffusion. The difference between diffusion rate and crystallization induces crystallization front instabilities, and a characteristic length $\delta = v_{\mathrm{d}}/v_{\mathrm{c}}$ (also called Keith–Padden parameter) defines the scale of this instability, which is effectively the distance over which impurities are excluded from the growth front^60,61^. This condition marks up an important situation where BHJ thin film should be post-treated, that an interconnected crystalline domain forms one phase within a general mixed region to perform the charge separation and carrier transport function. It should be noted that the crystallization-induced phase separation leads to gradually elevated *T*_g_ in mixing region due to enrichment of high *T*_g_ component, forming a system similar to bijels that lock the morphology with limited length scale of phase separation^45^. Further raising the *T*_c_, the high diffusion rate of the amorphous component leads to quick permeation that a uniform component distribution exists in mixed region, and the exact content depends on crystallinity (Region III). As mentioned above, the average composition profiles of the mixed region under Region II and III will be different from the initial compositions, the *ν*_c_ will change instead of the being constant as in Region I. As shown from the Eq. (5), *F*_n_, *F*_e_, and *F*_v_ are all composition dependent. It has shown that the deceleration of crystallization in Regime II is mainly caused by slowing down of the molecular motion by approaching the glass transition temperature, i.e., by vitrification (*F*_v_), and only to a small extent to dilution effect (*F*_e_)^56^. The deceleration of crystallization can also happen in the Regime III but minor in effect^53^.

Returning to OPV blends, the optimized annealing temperature is usually at an intermediate level ranging from 80 to 120 °C. There are reports shows that the concentration profile is non-uniform from crystal growth front to mixed region^62^. Thus, thermal annealing process falls into Region II. This is the case for DR3TSBDT:PC_71_BM and DTS(PTTh_2_)_2_:PC_71_BM and many other high-performance OPV systems^63,64^. The PCBM has a *T*_g_ of 120–160 °C, and the investigated donor materials have *T*_g_ between 25–70 °C (Supplementary Figs. 13–15)^40^. Thus, a binary mixture should have a compositional dependent *T*_g_ that is around 70–90 °C in as-cast thin film using Fox equation for estimation^65^. The as-cast DR3TSBDT:PC_71_BM film shows moderate crystallinity, thus the PC_71_BM concentration in the mixed region is higher than the blending ratio. When thermally annealed at 100 °C, the crystallites within the donor-rich region continue to grow and PC_71_BM get expelled to the mixed region and get stabilized within 10 min due to the vitrification of the interface and the mixed region upon PC_71_BM enrichment. The as-cast DTS(PTTh_2_)_2_:PC_71_BM film is an amorphous mixture with no phase separation. The features of crystallization and phase separation begin to show up at 70 °C as seen from in situ data, indicating that the thin film *T*_g_ is around this temperature. The deceleration of crystallization is seen in DR3TSBDT:PC_71_BM and DTS(PTTh_2_)_2_:PC_71_BM, as expected from the above theoretical discourses. Under higher annealing temperature (140 °C for the two systems under investigation), distinctive decoupling of crystallization and phase separation shows up, as seen from the quickly enlarged differences between CCL and phase separation (Fig. 4). The driving force of phase separation above this temperature is beyond crystallization. A plausible reason is that the system goes into the miscibility gap and SD starts to dominate the morphology evolution (route B in Fig. 5b).

**Fig. 6:**
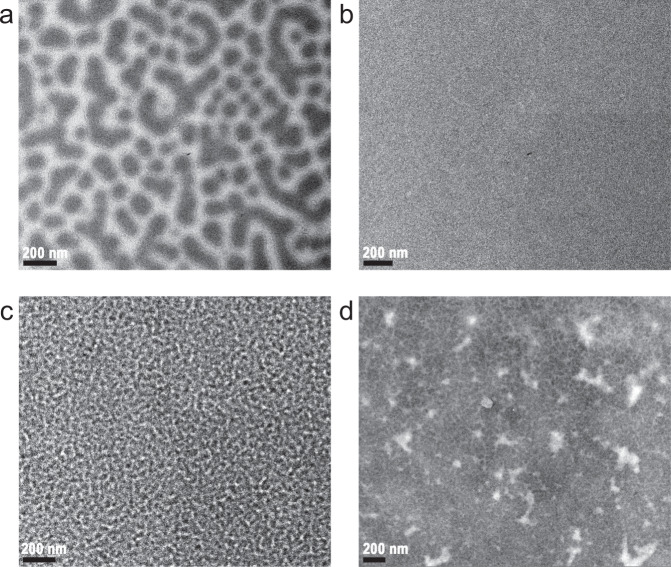
Transmission electron microscopy (TEM) images. Morphology of DPPEZnP-TEH:PC_61_BM **a** as-cast thin film, **b** pyridine additive processed thin film, **c** pyridine additive processed thin film after thermal annealing, and **d** pyridine and 1,8-diiodooctane (DIO) dual additive processed film after thermal annealing. Adapted with permission from ref. ^20^ and ref. ^21^. Copyright (2015) American Chemical Society and (2016) WILEY-VCH Verlag GmbH & Co. KGaA, Weinheim.

DPPEZnP-TEH:PC_61_BM is quite different from the above discussed situation. DPPEZnP-TEH has a weaker crystallinity compared to DR3TSBDT and DTS(PTTh_2_)_2_ and adopts a larger *χ* with PC_61_BM. The as-cast film state was confirmed with large size phase separation due to the strong demixing tendency (Fig. 6a). Such morphology is quickly developed and remains stable at high temperature up to 200 °C due to the pinning effect^54,55^. It is interesting to note that adding 1% pyridine in film processing can drastically change the kinetic route during film formation that leads to a homogeneous mixed state of two immiscible materials (Fig. 6b)^66,67^. Thus, related discussions need to consider the glass transition line, miscibility gap, and melting temperature line, where the coupling and competition of crystallization and demixing come into play^60,61^. When two or more non-equilibrium phenomena take place simultaneously, the final morphology depends strongly on the complicated dynamics, and the equilibrium phase diagram ceases to function. Multi-length scale morphology is usually obtained (Fig. 6c, d)^21^, regardless of whether the system adopts a miscibility gap with lower critical solution temperature (LCST) or upper critical solution temperature (UCST). The coupling and competition of crystallization and demixing at specific annealing temperature can be complicated. Some possible kinetic pathways are labeled as C, D, and E using UCST for demonstration (shown in Fig. 5c). (1) Route C represents simultaneous spinodal decomposition (SD) and crystallization, in which the system goes above the *T*_g_ line in the unstable region of the miscibility gap quickly decomposes and crystallizes; (2) Route D represents simultaneous binodal decomposition (BD) and crystallization in the metastable region; (3) Route E represents crystallization-induced demixing outside the miscibility gap. For DPPEZnP-TEH:PC_61_BM system, the large mismatch of CCL and phase separation distance indicates that the primary driving force for phase separation is demixing. Even if pyridine is used to form a uniform mixture, the strong tendency of demixing coupled with crystallization leads to multi-length scaled phase separation in which the small crystallization cannot be observed (Fig. 6c)^21^. An even more complicated recipe of adding pyridine and 1,8-diiodooctane (DIO) dual additives leads to more complicated morphology formation, in which DIO could induce PC_61_BM aggregation, and thus a more complicated multi-length scaled morphology can be obtained (Fig. 6d). All these pathways have to pass the *T*_g_ line to get enough molecular mobility.

### Morphology at interface

**Fig. 7:**
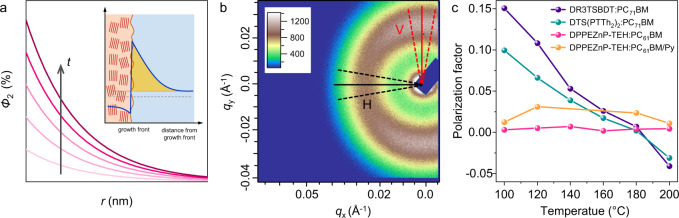
Bulk heterojunction interfacial properties. **a** Schematic PCBM concentration (*Φ*_2_) profiles as a function of distance from the growth front (*r*) and time (*t*). Inset is composition profiles at growth front of Region II. **b** Resonant soft X-ray scattering (RSoXS) 2D image of DR3TSBDT:PC_71_BM under 100 °C annealing. The area between dashed line around horizontal direction (H) and vertical direction (V) indicated by solid black and red line, respectively are used to calculate polarization factor. **c** Polarization factor change with different annealing temperatures.

Regarding the difference in morphology formation, the interfacial property is another important feature in addition to length scale of phase separation. In the RSoXS experiments under different annealing temperatures (shown in Fig. 4), we see the slope of the scattering profile in high *q* region decreases for DR3TSBDT:PC_71_BM and DTS(PTTh_2_)_2_:PC_71_BM blends upon increasing annealing temperature, which indicates the interfacial width at phase separated frontier get coarsened. In DPPEZnP-TEH:PC_61_BM/Py blends, the high *q* region slope increases at higher temperatures. Thus, in OPV the crystallization-induced phase separation leads to rougher interface while the decomposition driven phase separation induces sharper interface. OPV donor materials show preferred orientation quite common in varied systems. Such properties in associating with crystal growth induce crystallization frontier molecular orientation that accelerates charge separation. The general materials’ distribution profile upon Region II crystallization is shown in Fig. 7a^68^. Moreover, RSoXS scattering anisotropy is seen for DR3TSBDT:PC_71_BM blend thin film (Fig. 7b), representing a preferential face-on interfacial orientation that benefits charge separation^25,69,70^. It is seen that under a mild temperature annealing as in Region II, scattering anisotropy is retained, so does interfacial orientation. In high-temperature annealing (Region III) where the crystal growth becomes fast but less uniform, and demixing starts to dictate thin-film morphology. Disordered interfacial orientation is expected, and scattering becomes isotropic (Fig. 7c). Summarizing scattering anisotropy of all four cases reveals an interesting feature that interfacial orientation is preserved in crystallization-induced phase separation domain, which loses correlation in SD-induced phase separation. The unfavorable length scales and interfacial orientation result in poor device performances in solar cell devices.

### Linking morphology with device performance

**Fig. 8:**
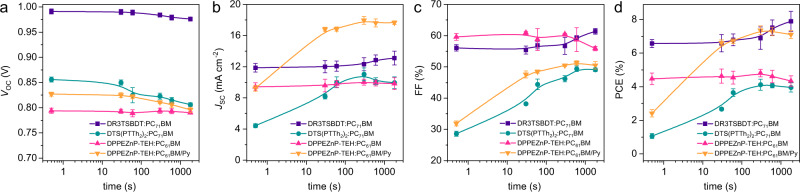
Device performance change. Change of **a** *V*_OC_, **b** *J*_SC_, **c** FF, and **d** PCE of DR3TSBDT:PC_71_BM, DTS(PTTh_2_)_2_:PC_71_BM, DPPEZnP-TEH:PC_61_BM and DPPEZnP-TEH:PC_61_BM/Py under different thermal annealing time (error bars represent the standard deviation over ten devices).

The morphology investigation and device performance correlations are examined. OPV device performances under different annealing time are summarized in Supplementary Table 2. Mild temperature annealing (100–110 °C) is used to drive the phase separation. It is seen that as-cast DR3TSBDT:PC_71_BM device shows good performance because interpenetrating network morphology has already formed during solvent drying. DTS(PTTh_2_)_2_:PC_71_BM and DPPEZnP-TEH:PC_61_BM/Py that are well mixed in as-cast condition show quite low performances. Temporal change of device performances under annealing are shown in Fig. 8. Quick jumping of *J*_SC_ and FF is seen for DTS(PTTh_2_)_2_:PC_71_BM and DPPEZnP-TEH:PC_61_BM/Py within 1 min of annealing. Such a change correlates well with the annealing process that the BHJ bicontinuous morphology is formed, and high specific inner surface area and transporting pathways are established. Further annealing within the BHJ framework improves thin-film crystallinity and phase purity of mixed domain, and slow increases in *J*_SC_ and FF are recorded, as seen from DR3TSBDT:PC_71_BM, DTS(PTTh_2_)_2_:PC_71_BM and DPPEZnP-TEH:PC_61_BM/Py. Such results demonstrate a good correlation between device performance and morphology details, and more importantly can reveal the fundamental aspects of morphology formation mechanism.

### Extending the observation to other systems

A retrospective analysis is paid to the extensively studied P3HT:PC_61_BM blends to validate the generality. P3HT and PC_61_BM are highly miscible^40,71–73^, and they also adopt a eutectic feature belonging to the situation of Fig. 5a^74^. It has been shown that the onset annealing temperature (representing *T*_g_) for improved device efficiency increases with increasing PC_61_BM wt%. The best performance is achieved in the system with 40 wt% PC_61_BM under 140–150 °C annealing^73^. These findings fall in the above discussed theory framework of using controlled crystallization to optimize the morphology. The eutectic feature of this system shifts away from conventional phase separation pathways, and the condition to facilitate P3HT crystallization while keeping the mixed region homogeneous is the key, which falls into Region II and III.

Further discussion is extended to the NFA OPV blends. Thermal annealing at mild temperature is commonly used to enhance device performances, even from barely working to remarkable efficiencies^75^, which also follows the crystallization-induced phase separation mechanism in Region II. It should be noted that in NFA blends both donor and acceptor materials are semi-crystalline, and thus the discourse needs to take both crystallization processes into account.

**Fig. 9:**
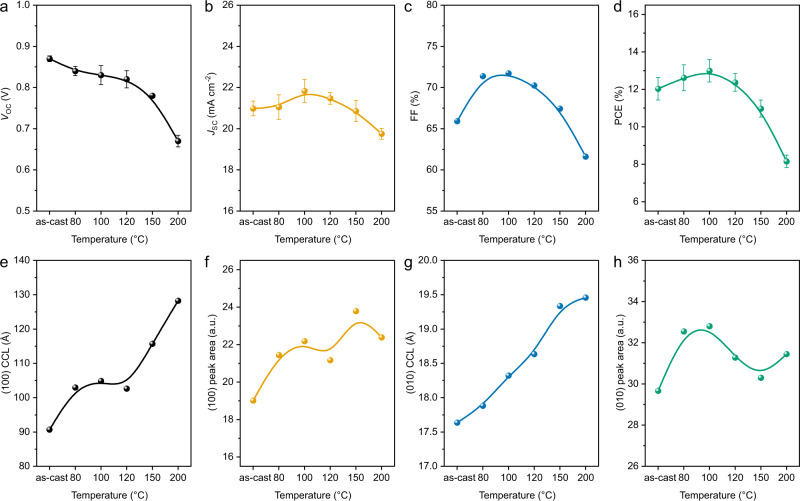
Device performance and morphology parameters of PM6:ITIC-4F. PM6:ITIC-4F device performances of **a** *V*_OC_, **b** *J*_SC_, **c** FF, and **d** PCE (error bars represent the standard deviation over ten devices), and related film morphology parameters of **e** (100) CCL, **f** (100) peak area, **g** (010) CCL, and **h** (010) peak area under different annealing temperature.

We look further into high-efficiency semi-crystalline conjugated polymer and NFA blends. PBDB-T:ITIC, PCE10:IEICO-4F, PM6:Y6 and PM6:IT-4F blends are chosen as model systems. BHJ thin films were annealed at different temperatures for 10 min to manipulate the morphology. As seen from these device performances, all systems show a performance increase under specific thermal stress and then bear gradual performance attenuation at higher temperature annealing (Fig. 9 and Supplementary Figs. 22–24). Taking PM6:ITIC-4F system as an example, a quite steady *J*_SC_ is seen under all annealing temperatures, and FF displays the best value at 80 °C (Fig. 9b, c). RSoXS shows that phase separation of PM6:ITIC-4F remains almost unchanged using different annealing temperature (Supplementary Fig. 16). The CCL of PM6 (100) peak at *q* ≈ 0.3 Å^−1^ is found to continuously increase from 90 Å (as-cast) to 128 Å (200 °C). The cases of PM6:ITIC-4F are similar to the DR3TSBDT:PC_71_BM blends discussed above. Therefore, the intermediate temperature annealing is beneficial that optimizes crystalline morphology without getting into miscibility gap.

## Discussion

To sum up, we studied the morphology and its evolution mechanism of representative OPV systems with observations that can be applied to understand the morphology formation in most OPV blends. The selected systems with different crystallization and phase separation features cover the most cases in OPV research, and thus of general interest. It is clearly seen that the non-equilibrium morphology formed via fast spin coating yields different morphologies that cannot be directly described and predicted by Flory-Huggins interaction parameter, which is more valuable in predicting equilibrium state phase separation as commonly seen in block copolymer systems. Detailed in situ and ex situ characterizations on BHJ thin films reveal the importance of coupling and competition of crystallization and demixing in determining the morphology of BHJ thin film. The general phase separation (either by demixing or crystallization) can happen if a blend endures a thermal stress over *T*_g_ and does not necessarily to get into the miscibility gap. A crystallization-induced phase separation is expected above film *T*_g_ and outside the miscibility gap, which decouples crystallization from demixing. Such temperature region is critically important in OPV morphology optimization, in which we can manipulate crystallization process to control the length scale of phase separation, as well as the phase purity of mixed region and interfacial orientation between different phases. It is seen in devices that crystallization-induced phase separation can help to increase *J*_SC_ and FF by establishing a bicontinuous-interpenetrating network and purifying constituted phases. It is also observed that high-temperature annealing leads to rigorous phase separation via SD, which yields large phase separation with interface broadening and less orientation order. The current research established a more robust structure-property relationship that covers fundamental mechanism and application region. These findings based on the unified phase diagram framework can help understand the morphology transition details upon post-treatment, which is beneficial for clearing up confusions of understanding the complex multi-length scale morphology in the fields and provides useful to morphology optimization guidelines for designing novel OPV blends with improved device efficiency and stability.

## Methods

### Materials

The DR3TSBDT, DTS(PTTh_2_)_2_, and DPPEZnP-TEH were synthesized according to previous reports. PC_61_BM and PC_71_BM were purchased from Nano-C Inc. PBDB-T, PM6, PCE10, ITIC, ITIC-4F, IEICO, and Y6 are purchased from Solarmer Materials Inc. All the other reagents and chemicals were purchased from Sigma Aldrich or Acros and used as received.

### X-ray scattering characterization

Grazing incidence small- and wide-angle X-ray scattering (GISAXS/GIWAXS) measurements were conducted on beamline 7.3.3 at Advanced Light Source, Lawrence Berkeley National Laboratory. Samples were prepared on PEDOT:PSS modified Si substrates using identical conditions as those used in devices. The incident X-ray with wavelength of 1.240 Å (10 keV) passed through the samples at a grazing incidence angle of 0.16°, and the scattered X-ray was detected by a Dectris Pilatus 2 M photon counting detector. The sample detector distance for GIWAXS and GISAXS is around 300 mm and 3.5 m separately using Ag behenate for refinement. The beam size is approximately 250 × 150 μm. For in situ experiments, a heating stage was installed in helium box to achieve precise substrate temperature control under real time measurements. The onset temperature was 40 °C with a heating rate of 20 °C min^−1^ and then stabilized at the target annealing temperature. Scattering data was collected every 30 s per frame with 2 s exposure time. Resonant soft X-ray scattering (RSoXS) measurements in transmission mode were performed on beamline 11.0.1.2 using 284.2 eV photon energy at Advanced Light Source, Lawrence Berkeley National Laboratory. Samples for RSoXS measurements were prepared on a PEDOT:PSS modified Si substrate under the same conditions as those used for device fabrication, and then transferred by floating in water to a 1.5 × 1.5 mm, 100 nm thick Si_3_N_4_ membrane supported by a 5 × 5 mm, 200 μm thick silicon nitride window that obtained from CleanSiN. Two-dimensional (2D) scattering patterns were collected on an in-vacuum CCD camera (Princeton Instrument PI-MTE).

### Glass transition temperature measurements

Differential scanning calorimetry (DSC) measurements were carried out on a Mettler Toledo DSC 3+ equipped with FRS 6+ sensor and Huber TC 100 intracooler, using Al light 20 µl crucible. A heat-cool-heat cycle with heating/cooling rate of 10 °C min^−1^ was applied in the temperature of 0 to 300 °C (except for DR3TSBDT, the temperature range was −50 to 250 °C). To enhance the signal and better locate *T*_g_, a blank curve using empty crucible was performed and later subtracted from the sample heat flow, and *T*_g_ was determined using the half-step method.

### Melting point depression measurements

Melting point depression experiments was carried out using differential scanning calorimetry (DSC) to evaluate interaction parameter *χ* between high-crystalline small molecule and PC_71_BM. The samples were prepared by adding little amount of PC_71_BM into small molecule (2–25 wt %). These blend samples were dissolved in chloroform and then drop casted to DSC pan. Then, DSC (TA Instruments, Q2000) was to check the melting point change. The samples were firstly heated at a rate of 10 °C min^−1^ to 260 °C and held for 5 minutes. Subsequently the samples were cooled to 0 °C at 2 °C min^−1^ and held for 5 minutes. Then the samples were heated again at a rate of 10 °C min^−1^ to 260 °C, and the melting point (*T*_m_) was obtained from the high-temperature side intersection of the base line with the tangent to the endotherm.

### Device fabrication and characterization

The devices were fabricated on pre-patterned ITO glass substrates using an ITO/PEDOT:PSS/SM:fullerene/Al structure, except for DTS(PTTh_2_)_2_:PC_71_BM using a ITO/PFN/SM:fullerene/MoO_*x*_/Al structure. After ultrasonically cleaned in detergent solution, deionized water, acetone and isopropyl alcohol, the substrates were dried in nitrogen flow and treated by UV-Ozone for 15 min. A 30 nm PEDOT:PSS (Clevious P VP AI 4083 H. C. Stark, Germany) or 10 nm thick PFN was then spin-coated onto the ITO substrates. The substrate with PEDOT:PSS were baked at 150 °C for 15 min. The substrates were then transferred into nitrogen glove box. All the active films were spin-coated following the processing conditions shown in Supplementary Table 2. Annealing were applied subsequently. A 10 nm MoO_x_ layer (for DTS(PTTh_2_)_2_: PC_71_BM only) and a 100 nm Al layer were sequentially thermally evaporated through a shadow mask to define the active area of the devices (3.14 mm^2^) and form the top cathode. All device fabrication processes are carried out in a nitrogen glove box at room temperature. The PCE was determined from *J*–*V* curve measurements (using a Keithley 2400 Source Meter) under a 100 mW cm^−2^, AM 1.5G spectrum from a solar simulator (Oriel model 91192). Masks made using laser beam cutting technology to have a well-defined area of 3.14 mm^2^ were attached to define the effective area for accurate measurement. The solar simulator illumination intensity was determined using a monocrystal silicon reference cell (Hamamatsu S1133, with KG-5 visible color filter) calibrated by the National Renewable Energy Laboratory (NREL). The *J*–*V* curves were measured along the forward scan direction from −0.2 to 1.5 V or the reverse scan direction from 1.5 to −0.2 V, yielding identical results. The scan speed and dwell times were fixed at 0.015 V s^−1^ and 20 ms, respectively.

### Reporting summary

Further information on research design is available in the Nature Research Reporting Summary linked to this article.

## Supplementary information

Supplementary Information

Reporting Summary

Solar Cells Reporting Summary

## Data Availability

The data supporting the results and findings of this study are available from the corresponding authors upon reasonable request.
