# Supplementary material for: The coupling and competition of crystallization and phase separation, correlating thermodynamics and kinetics in OPV morphology and performances
Source: Nat Commun. 2021 Jan 12;12:332. doi: 10.1038/s41467-020-20515-3 (PMC7804468; doi:10.1038/s41467-020-20515-3)
Supplement: Supplementary file 1 — Supplementary Information [file 41467_2020_20515_MOESM1_ESM.pdf]

The coupling and competition of crystallization and phase separation, correlating thermodynamics and kinetics in OPV morphology and performances

Zaiyu Wang[1,2], Ke Gao[3,*], Yuanyuan Kan[3], Ming Zhang[1], Chaoqun Qiu[1], Lei Zhu[1], Zhe Zhao[1], Xiaobin Peng[4], Wei Feng[5], Zhiyuan Qian[6], Xiaodan Gu[6], Alex K.-Y. Jen[3,7,*], Ben Zhong Tang[2], Yong Cao[4], Yongming Zhang[1], Feng Liu[1,*]

[1] Frontiers Science Center for Transformative Molecules and In-situ Center for Physical Science, School of Chemistry and Chemical Engineering, Shanghai Jiao Tong University, Shanghai 200240, China. [2] Department of Chemistry, The Hong Kong University of Science and Technology, Clear Water Bay, Kowloon, Hong Kong 999077, China. [3] Department of Materials Science and Engineering and Department of Chemistry, University of Washington, Seattle, Washington 98195, USA. [4] State Key Lab of Luminescent Materials and Devices, South China University of Technology, Guangzhou 510640, China. [5] State Key Laboratory of Fluorinated Functional Membrane Materials, Shandong Dongyue Polymer Material Co. Ltd, Zibo, Shandong 256401, China. [6]School of Polymer Science and Engineering, Center for Optoelectronic Materials and Devices, The University of Southern Mississippi, Hattiesburg, MS, 39406, USA. [7] Department of Chemistry, City University of Hong Kong, Kowloon, Hong Kong 999077, China. These authors contribute equally: Zaiyu Wang, Ke Gao. * Correspondence should be addressed to K.G. (email: kegao@uw.edu) or to A.K.Y.J. (email: alexjen@cityu.edu.hk) or to F.L. (email: fengliu82@sjtu.edu.cn).

Table of Contents

Supplementary Note 1. Materials

Supplementary Note 2. X-ray scattering characterization and analysis

Supplementary Note 3. Morphology related terminology

Supplementary Note 4. Glass transition temperature measurement

Supplementary Note 5. Melting point depression

Supplementary Note 6. Device fabrication and performance characterization

Supplementary Note 1. Materials

The DR3TSBDT, $DTS(PTTh_2)_2$ and DPPEZnP-TEH were synthesized according to previous reports[1-3]. $PC_{61}BM$ and $PC_{71}BM$ were purchased from Nano-C Inc. PBDB-T, PM6, PCE10, ITIC, ITIC-4F, IEICO and Y6 are purchased from Solarmer Materials Inc. All the other reagents and chemicals were purchased from Sigma Aldrich or Acros and used as received.

Supplementary Table 1. Materials and corresponding chemical structures discussed in the main text.

Materials	Chemical structure
DR3TSBDT[1]	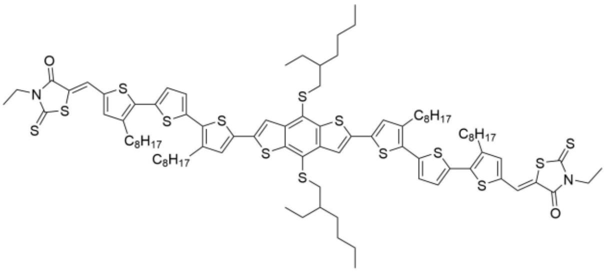
$DTS(PTTh_2)_2$[2]	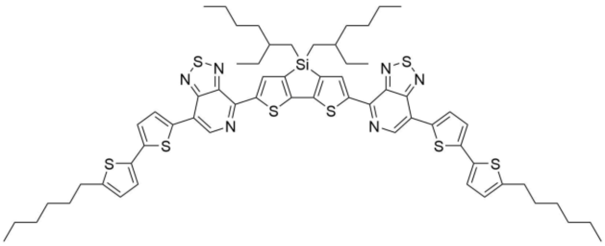
DPPEZnP-TEH[3]	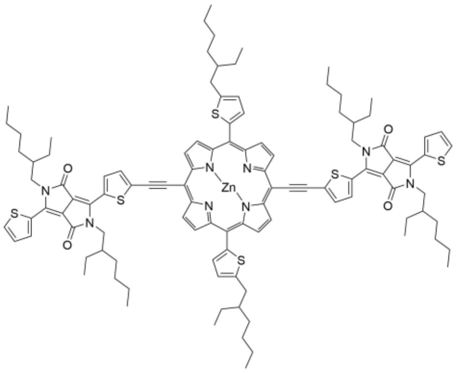
$PC_{61}BM$	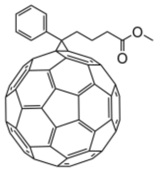
$PC_{71}BM$	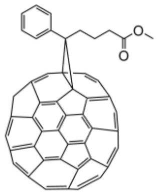

PBDB-T[4]	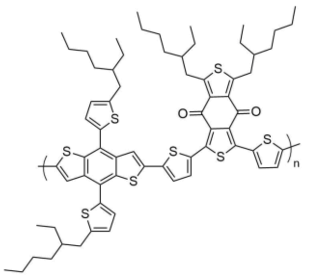
PM6[5]	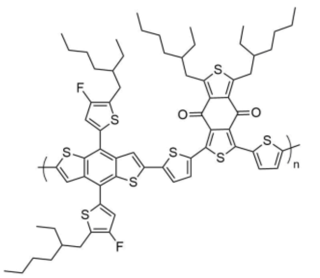
PCE-10[6]	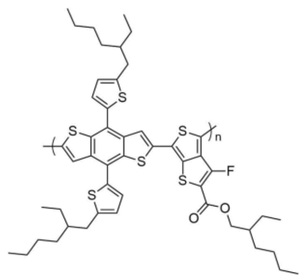
ITIC[7]	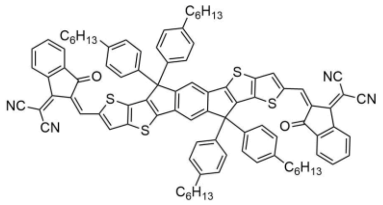
ITIC-4F[8]	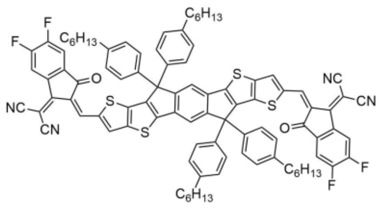
IEICO[9]	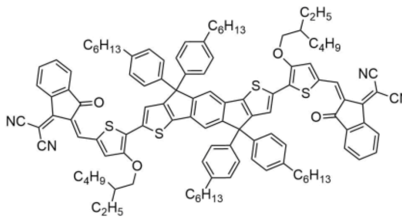
Y6[10]	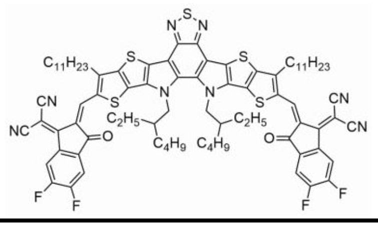

Supplementary Note 2. X-ray scattering characterization and analysis

Grazing incidence small- and wide-angle X-ray scattering (GISAXS/GIWAXS) measurements were conducted on beamline 7.3.3 at Advanced Light Source, Lawrence Berkeley National Laboratory[11]. Samples were prepared on PEDOT:PSS modified Si substrates using identical conditions as those used in devices. The incident X-ray with wavelength of 1.240 Å (10 keV) passed through the samples at a grazing incidence angle of 0.16°, and the scattered X-ray was detected by a Dectris Pilatus 2M photon counting detector. The sample detector distance for GIWAXS and GISAXS is around 300 mm and 3.5 m separately using Ag behenate for refinement. The beam size is approximately 250 μm × 150 μm. For *in situ* experiments, a heating stage was installed in helium box to achieve precise substrate temperature control under real time measurements. The onset temperature was 40 °C with a heating rate of 20 °C/min and then stabilized at the target annealing temperature. Scattering data was collected every 30 s per frame with 2 s exposure time. *In situ* GISAXS and GIWAXS were done on separate films pieces of 10 mm × 5 mm taken from the same original sample. The scattering images were processed using Nika software package[12]. The 1D line profiles of GIWAXS are sector averaged profile of ±15° around in-plane and out-of-plane direction. Only in-plane *in situ* GIWAXS profiles are presented to compare with the *in situ* in-plane GISAXS profiles shown in Fig. 1.

Resonant soft X-ray scattering (RSoXS) measurements in transmission mode were performed on beamline 11.0.1.2 using 284.2 eV photon energy at Advanced Light Source (ALS), Lawrence Berkeley National Laboratory[13]. Samples for RSoXS measurements were prepared on a PEDOT:PSS modified Si substrate under the same conditions as those used for device fabrication, and then transferred by floating in water to a 1.5 mm × 1.5 mm, 100 nm thick Si_3N_4 membrane supported by a 5 mm × 5 mm, 200 μm thick silicon nitride window that obtained from CleanSiN. 2D scattering patterns were collected on an in-vacuum CCD camera (Princeton Instrument PI-MTE). The sample detector distance was calibrated from diffraction peaks of a triblock copolymer poly(isoprene-b-styrene-b-2-vinyl pyridine), which has a known spacing of 391 Å. The beam size at the sample is approximately 100 μm by 200 μm.

Gaussian multipeak fitting was used to fit the (100) peak to get the peak position, intensity and full width at half maximum (FWHM). Packing distance d of (100) peak can be calculated using $d = 2\pi/q$, where q is the peak position. Scherrer equation (Supplementary Equation (1)) is used to calculate the crystal coherence length (CCL) or crystal size[14]:

$$CCL = \frac{2\pi K}{\Delta q} \quad (1)$$

where Δq is FWHM in reciprocal space and Scherrer constant K is often reported to be ~0.9 derived from the estimation $K = 2[2\ln(2)/\pi]^{1/2} \cong 0.93$ from Scherrer's original paper[15].

Paracrystallinity g-factor (Supplementary Fig. 4) representing accumulative structural disorder was calculated using the following equation[16,17]:

$$g = \sqrt{\frac{\Delta q}{2\pi q}} \quad (2)$$

According to the g-factor analysis (shown in Supplementary Fig. 4), all of these semicrystalline materials are at best paracrystalline (for DR3TSBDT and $DTS(PTTH_2)_2$) and mostly amorphous (for DPPEZnP-TEH) in the blends. For purposes of this study and comparisons of the 4 donor/acceptor blends considered, we refer to the DR3TSBDT blend sample as "highly crystalline" and DPPEZnP-TEH as "moderate" in the main text; however, it should make it clear that these are all relative terms for comparing the samples in this study. None of the samples are considered crystalline materials.

Degree of crystallinity is another useful morphological parameter to give an accurate estimation of thin film crystallinity based on analyzing the full pole figure of GIWAXS 2D patterns. However, since we try to connect crystallization and phase separation information together in our case, the material crystallization in in-plane direction is more concerned and we did not carry out the calculation of degree of crystallinity. The audience can read related references for further exploration on this topic[18,19].

The correlation length model and the broad peak model are employed to fit the GISAXS data. Correlation length model (Supplementary Equation (3)) is commonly used for small angle scattering (SAS) data characterized a low q signal and a high q signal[20]. The two scale factors A and C, the incoherent background B and the two exponent factors n and m are used as fitting parameters. The parameter ξ is correlation length (length beyond which correlations die out).

$$I(q) = \frac{A}{q^n} + \frac{C}{1 + (q\xi)^m} + B \quad (3)$$

The broad peak model (Supplementary Eq. (3)) calculates an empirical function form for SAS data characterized by a broad scattering peak. This model is based on the correlation length model with q in the second term replaced by $|q - q_0|$. Many SAS spectra are characterized by a broad peak even though they are from amorphous soft materials. The d-spacing corresponding to the broad peak is a characteristic distance between the scattering inhomogeneities, and so we refer to it as the "phase separation peak distance", or for brevity as the "phase separation". The scattering intensity $I(q)$ is calculated as

$$I(q) = \frac{A}{q^n} + \frac{C}{1 + (|q - q_0|\xi)^m} + B \quad (4)$$

here the peak position is related to the d-spacing as $q_0 = 2\pi/d_0$.

Most GISAXS profiles are fitted to the broad peak model (Supplementary Equation (4)), except the data where there is no visible peak in the measured region were fitted using correlation length model (Supplementary Equation (3)). Meanwhile, Porod exponents (representing fractal dimension) were also determined by fitting the high q region where intensity decay follows a Porod law. A fitting demonstration is shown in Supplementary Fig. 5 to show the accuracy of the fitting model.

Interfacial polarization factor representing interfacial orientation is determined by the ratio of sector averaged intensity in vertical and horizontal direction of RSoXS 2D image as shown in Fig. 7b.

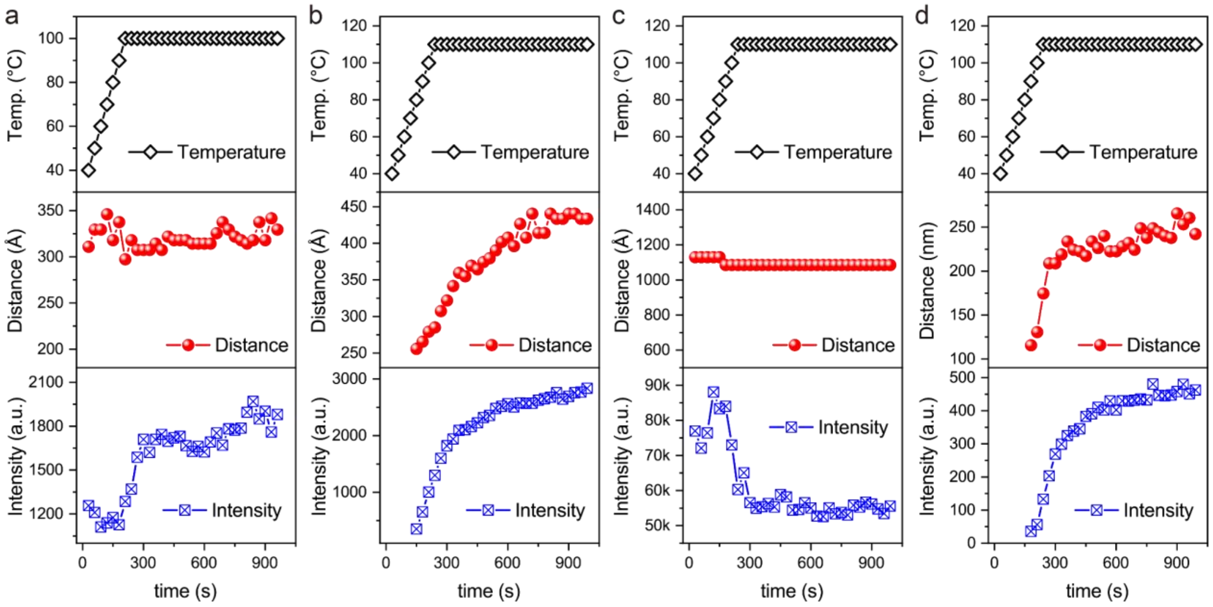

Supplementary Fig. 1 Heating temperature, phase separation peak distance (direct conversion from peak location q) and directing reading peak intensity as function of time (30 s per frame) of (**a**) DR3TSBDT:$PC_{71}BM$, (**b**) DTS($PTTh_2$)$_2$:$PC_{71}BM$, (**c**) DPPEZnP-TEH:$PC_{61}BM$ and (**d**) DPPEZnP-TEH:$PC_{61}BM$/Py during *in situ* GISAXS experiments (These quantities are also overlaid in Fig. 3 in the main text.)

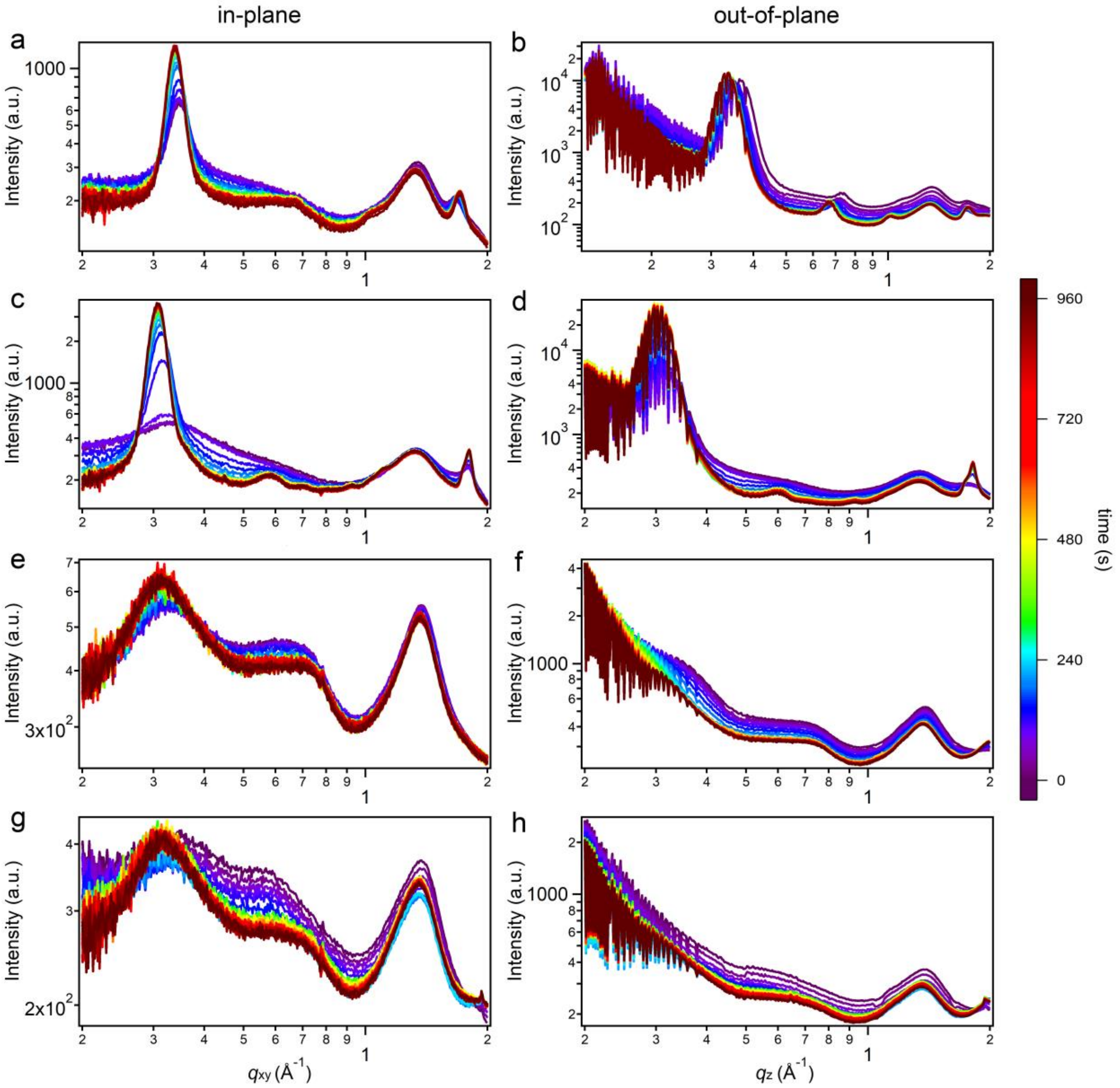

Supplementary Fig. 2 *In situ* GIWAXS in-plane and out-of-plane profiles of (**a**, **b**) DR3TSBDT:$PC_{71}BM$, (**c**, **d**) DTS$(PTTh_2)_2$:$PC_{71}BM$, (**e**, **f**) DPPEZnP-TEH:$PC_{61}BM$ and (**g**, **h**) DPPEZnP-TEH:$PC_{61}BM$/Py

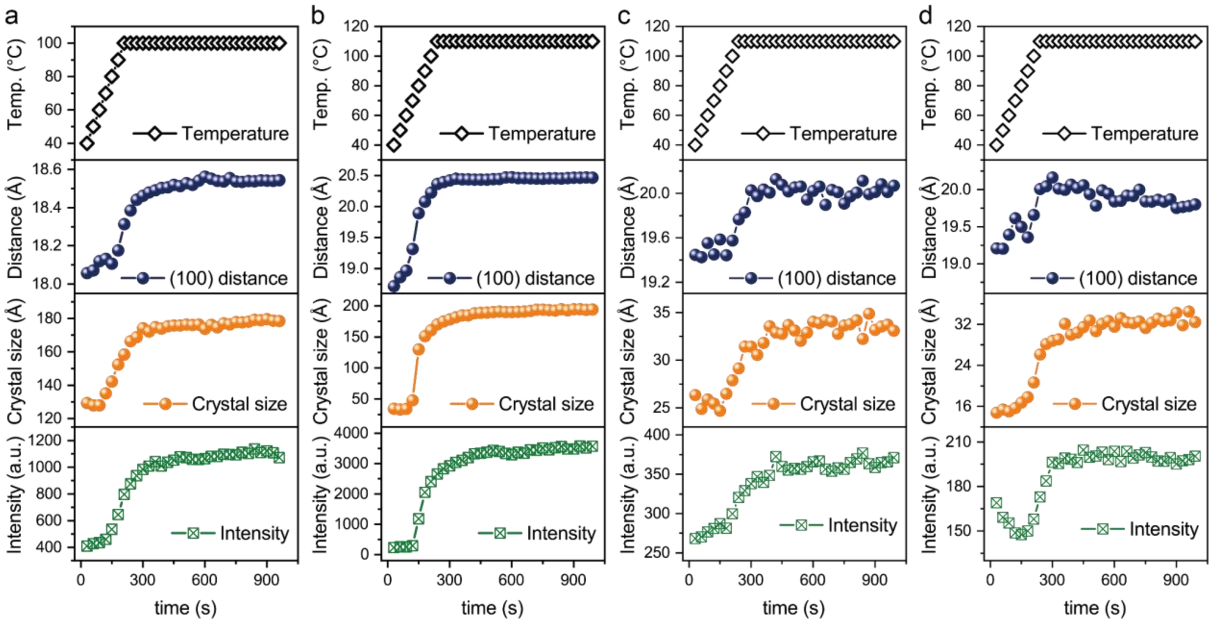

Supplementary Fig. 3 Heating temperature, in-plane (100) peak packing distance, crystal size along (100) direction and peak intensity as a function of time (30 s per frame) of (**a**) DR3TSBDT:$PC_{71}BM$, (**b**) DTS($PTTh_2$)$_2$:$PC_{71}BM$, (**c**) DPPEZnP-TEH:$PC_{61}BM$ and (**d**) DPPEZnP-TEH:$PC_{61}BM$/Py during *in situ* GIWAXS experiments (These quantities are also overlaid in Fig. 3 in the main text.)

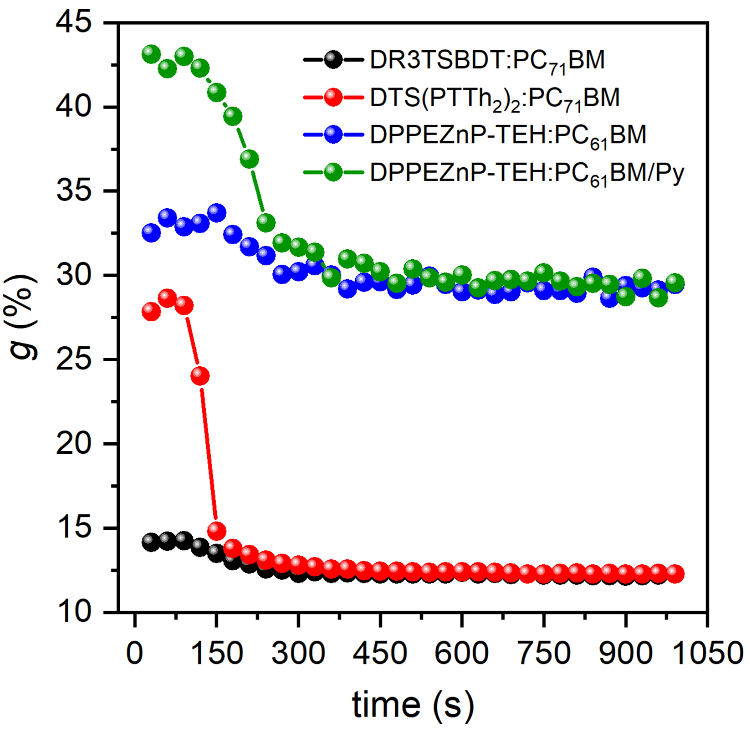

Supplementary Fig. 4 *g*-factor of DR3TSBDT:$PC_{71}BM$, DTS($PTTh_2$)$_2$:$PC_{71}BM$, DPPEZnP-TEH:$PC_{61}BM$ and DPPEZnP-TEH:$PC_{61}BM$/Py during *in situ* GIWAXS experiments.

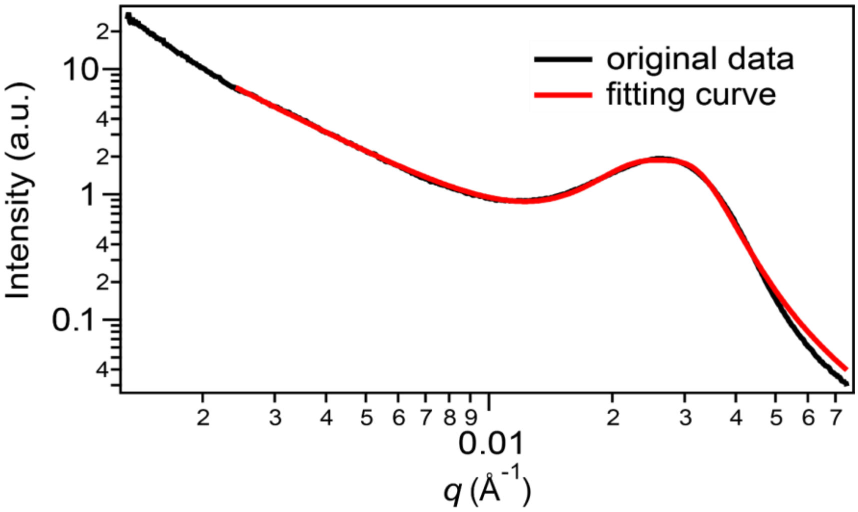

Supplementary Fig. 5 Demonstration of SAXS model fitting using broad peak model of data from Fig. 4a.

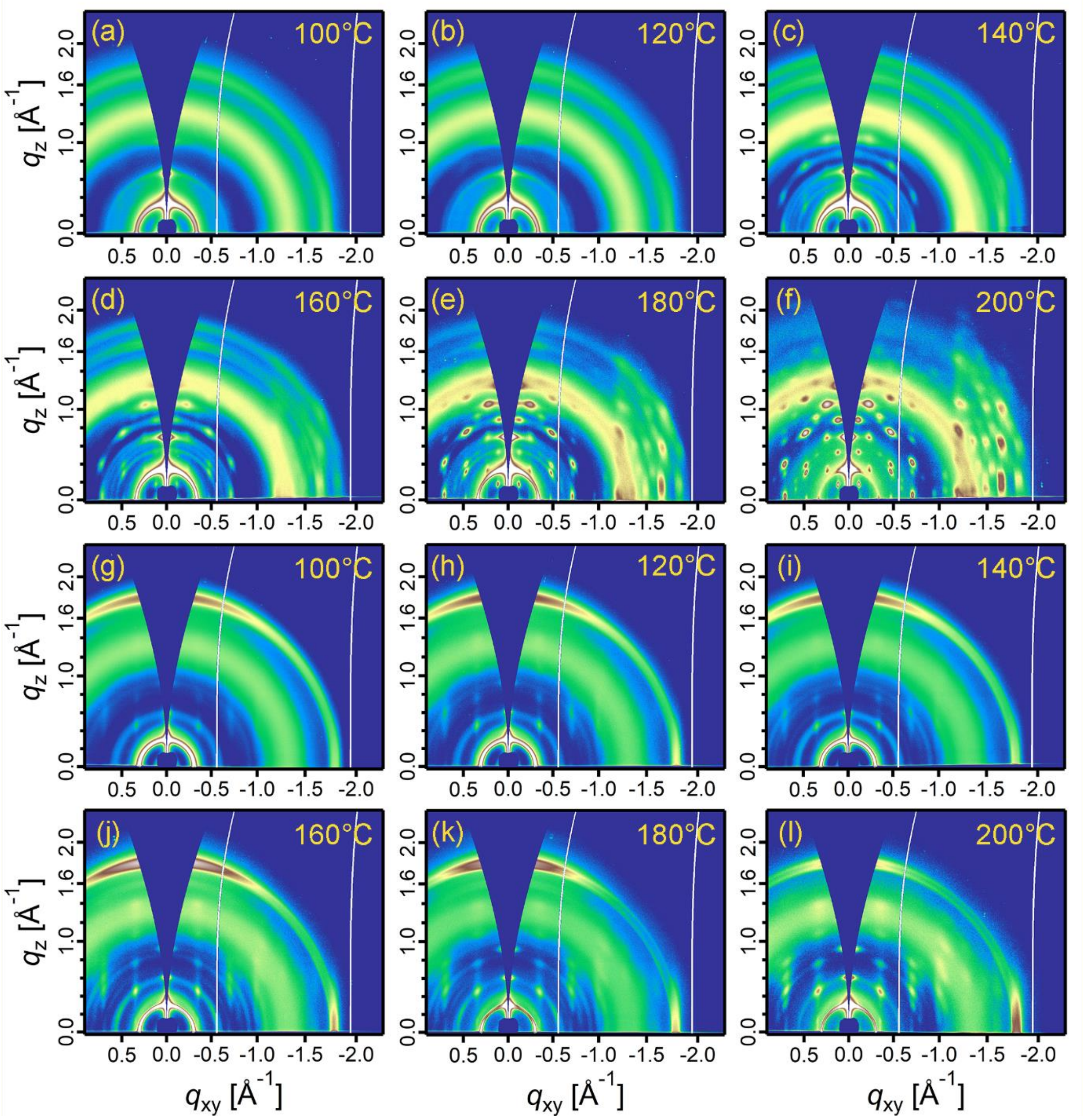

Supplementary Fig. 6. Static GIWAXS 2D patterns of (**a**-**f**) DR3TSBDT:$PC_{71}BM$ and (**g**-**l**) DTS($PTTh_2$)$_2$:$PC_{71}BM$ film at different annealing temperatures for 10 min.

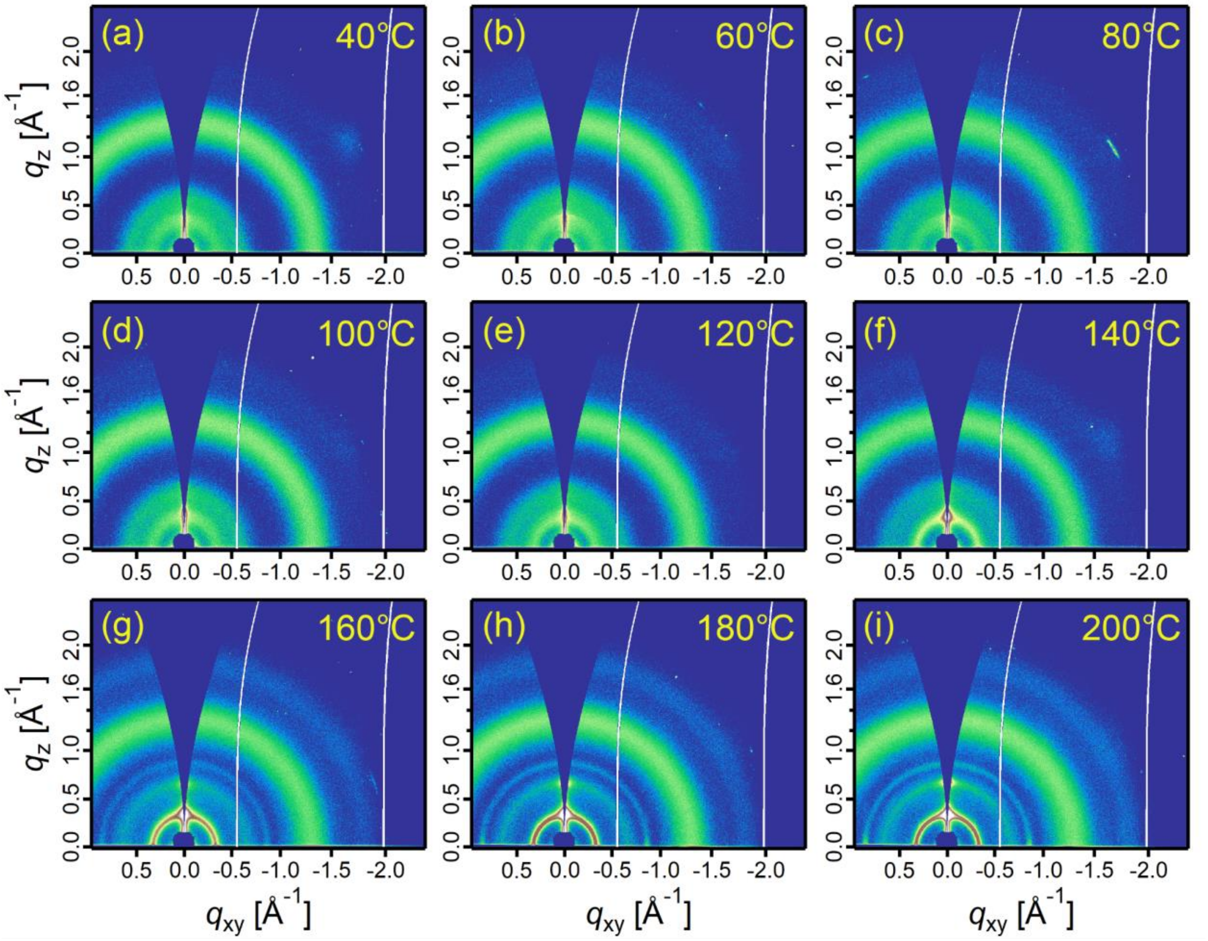

Supplementary Fig. 7 Static GIWAXS 2D patterns of DPPEZnP-TEH:$PC_{71}BM$ film at different annealing temperatures for 10 min.

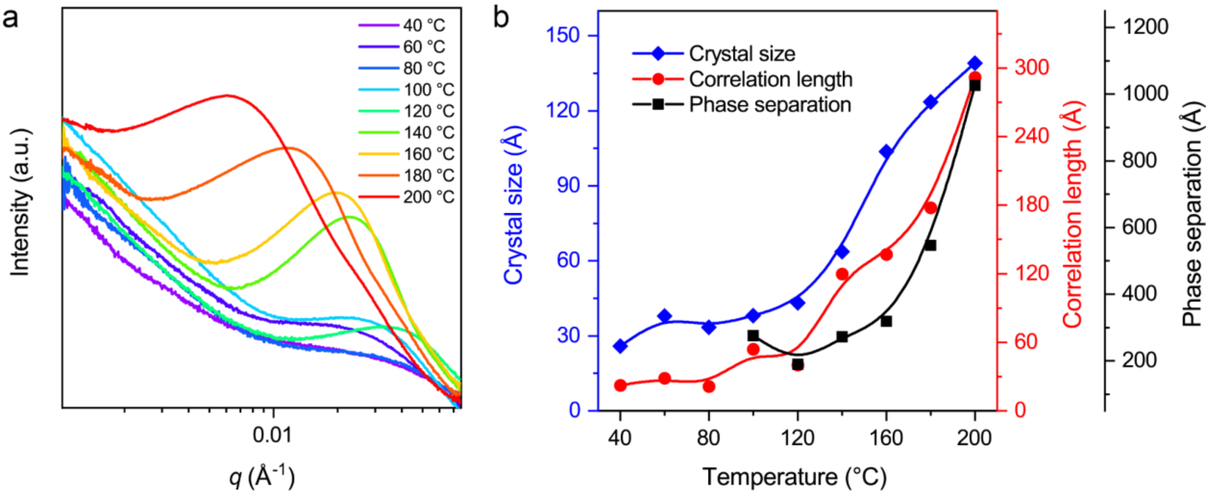

Supplementary Fig. 8 (a) RSoXS profiles and (b) morphological parameters (crystal size, correlation length and phase separation distance) of DPPEZnP-TEH:$PC_{71}BM$ at different annealing temperatures.

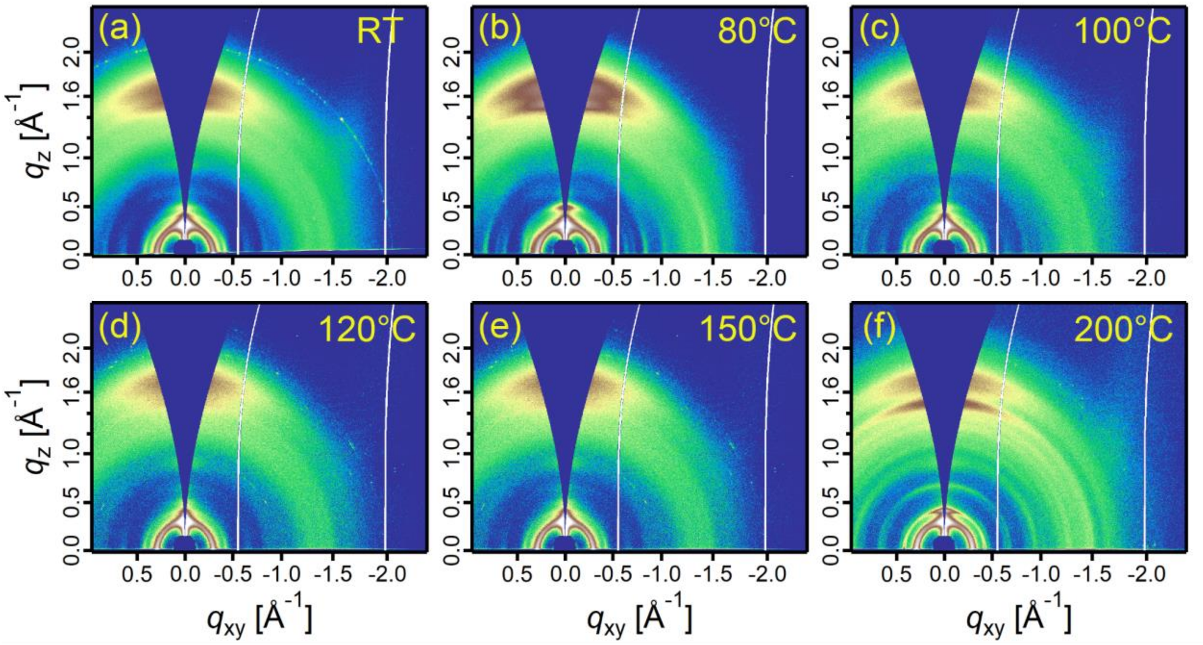

Supplementary Fig. 9 Static GIWAXS 2D patterns of (a-f) PBDB-T:ITIC at different annealing temperatures for 10 min.

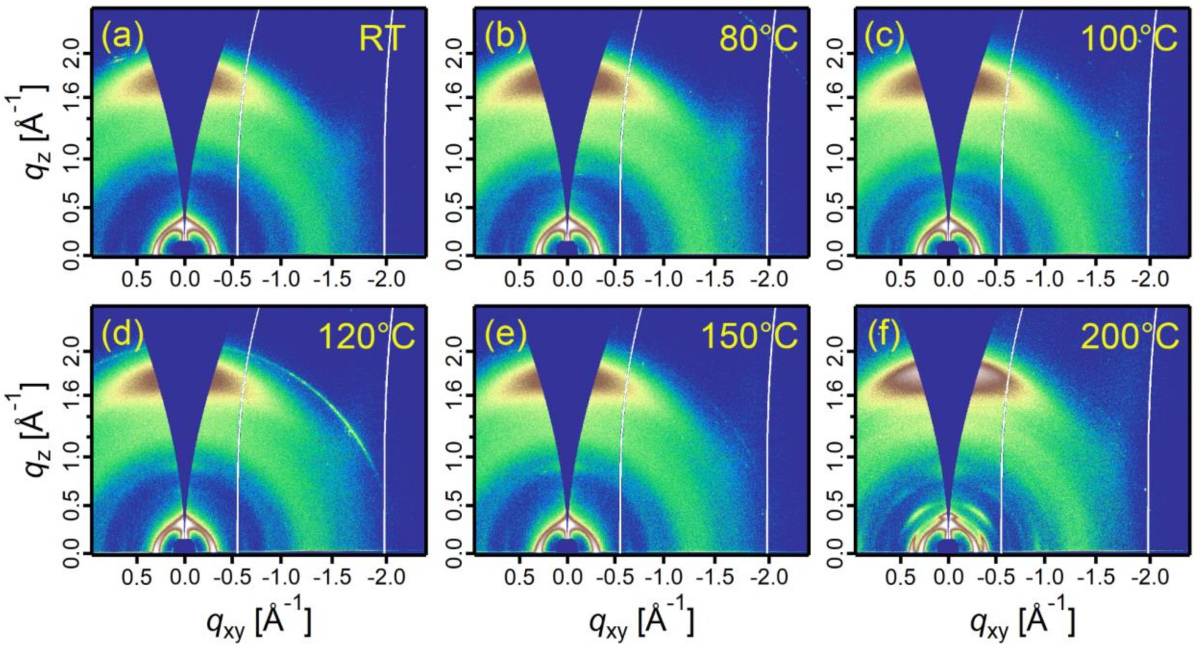

Supplementary Fig. 10 Static GIWAXS 2D patterns of (a-f) PM6:ITIC-4F at different annealing temperatures for 10 min.

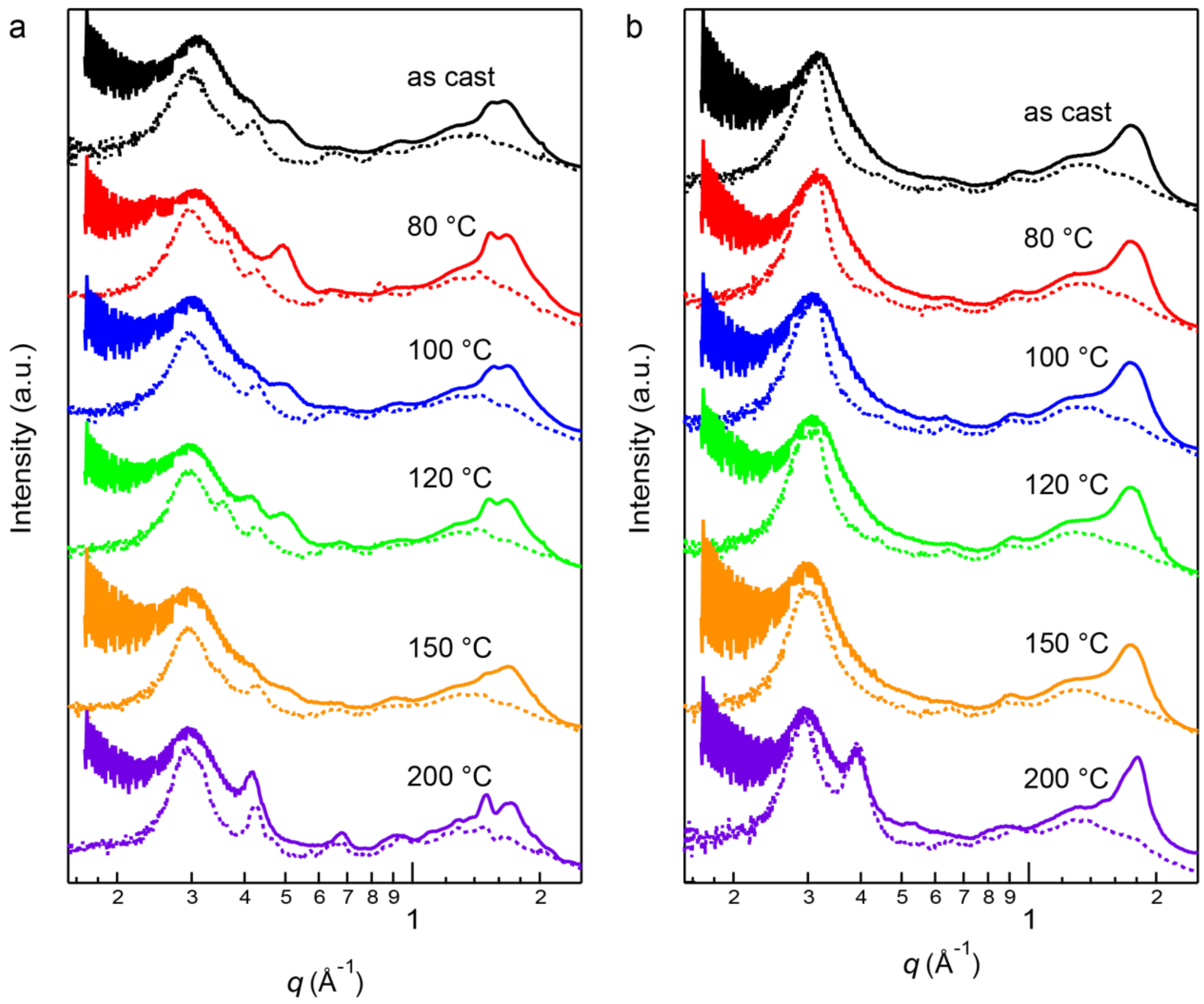

Supplementary Fig. 11 GIWAXS 1D line-cuts in in-plane (dashed line) and out-of-plane (solid line) direction of (a) PBDB-T:ITIC and (b) PM6:ITIC-4F at different annealing temperatures.

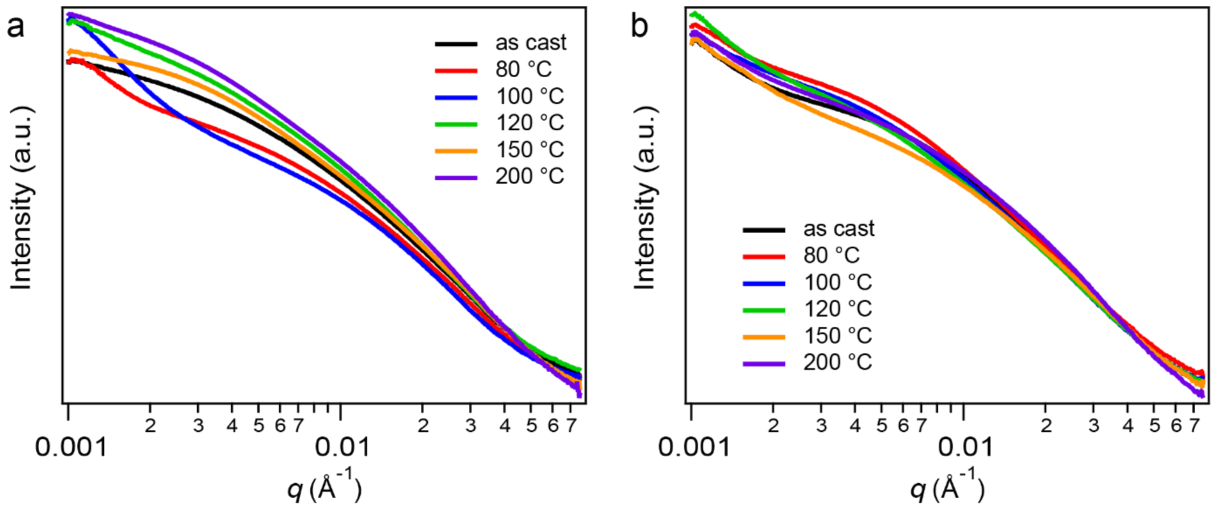

Supplementary Fig. 12 RSoXS profiles of (a) PBDB-T:ITIC and (b) PM6:ITIC-4F at different annealing temperatures.

Supplementary Note 3. Morphology Related Terminology

We clarify some basic and confusing terminologies involved in describing organic/polymer morphology by citing the term definition enacted by International Union of Pure and Applied Chemistry (IUPAC)[21-25], since universal adoption of a consensus on nomenclature is vital to the research community.

Miscibility: Capability of a mixture to form a single phase over certain ranges of temperature, pressure, and composition.

1. Whether or not a single phase exists depends on the chemical structure, molar-mass distribution, and molecular architecture of the components present.
2. The single phase in a mixture may be confirmed by light scattering, X-ray scattering, and neutron scattering.
3. For a two-component mixture, a necessary and sufficient condition for stable or metastable equilibrium of a homogeneous single phase is

$$\left(\frac{\partial^2 \Delta_{\mathrm{mix}} G}{\partial \phi^2}\right)_{T,p} > 0 \quad (5)$$

where $\Delta_{\mathrm{mix}}G$ is the Gibbs energy of mixing and ϕ the composition, where ϕ is usually taken as the volume fraction of one of the components. The system is unstable if the above second derivative is negative. The borderline (spinodal) between (meta)stable and unstable states is defined by the above second derivative equaling zero. If the compositions of two conjugate (coexisting) phases become identical upon a change of temperature or pressure, the third derivative also equals zero (defining a critical state).
4. If a mixture is thermodynamically metastable, it will demix if suitably nucleated. If a mixture is thermodynamically unstable, it will demix by spinodal decomposition or by nucleation and growth if suitably nucleated, provided there is *minimal kinetic hindrance*.
5. Miscibility is sometimes erroneously assigned on the basis that a blend exhibits a single T_g or optical clarity.

Metastable miscibility: Capability of a mixture to exist for an indefinite period of time as a single phase that is separated by a small or zero energy barrier from a thermodynamically more stable multiphase system.

1. Mixtures exhibiting metastable miscibility may remain unchanged or they may undergo phase separation, usually by nucleation or spinodal decomposition.
2. In polymers, because of the low mobility of polymer chains, particularly in a glassy state, metastable mixtures may exist for indefinite periods of time without phase separation. This has frequently led to confusion when metastable miscible polymer blends are erroneously claimed to be miscible.)

Compatibility: Capability of the individual component substances in either an immiscible polymer blend or a polymer composite to exhibit interfacial adhesion.

1. Use of the term "compatibility" to describe miscible systems is discouraged.
2. Compatibility is often established by the observation of mechanical integrity under the intended conditions of use of a composite or an immiscible polymer blend.

Flory-Huggins theory (Flory-Huggins-Staverman theory): Statistical thermodynamic mean-field theory of polymer solutions, formulated independently by Flory, Huggins, and Staverman, in which the thermodynamic quantities of the solution are derived from a simple concept of combinatorial entropy of mixing and a reduced Gibbs-energy parameter, the "χ interaction parameter".

χ interaction parameter: Interaction parameter, employed in the Flory-Huggins theory, to account for the contribution of the noncombinatorial entropy of mixing and the enthalpy of mixing to the Gibbs energy of mixing.

1. The definition and the name of the term have been modified to reflect its broader use in the context of polymer blends. In its simplest form, the χ parameter is defined according to the Flory-Huggins equation for binary mixtures for a mixture of amounts of substance n_1 and n_2 of components denoted 1 and 2, giving volume fractions ϕ_1 and ϕ_2, with the molecules of component 1 each conceptually consisting of x_1 segments whose Gibbs energy of interaction with segments of equal volume in the molecules of component 2 is characterized by the interaction parameter χ.
2. The χ interaction parameters characterizing a given system vary with composition, molar mass, and temperature.
3. B is an alternative parameter to χ, where $B = \chi RT/V_m$, in which V_m is the molar volume of one of the components of the mixture.

Morphology coarsening (phase ripening): Process by which phase domains increase in size during the aging of a multiphase material.

1. In the coarsening at the late stage of phase separation, volumes and compositions of phase domains are conserved.
2. Representative mechanisms for coarsening at the late stage of phase separation are: (1) material flow in domains driven by interfacial tension (observed in a co-continuous morphology), (2) the growth of domain size by evaporation from smaller droplets and condensation into larger droplets, and (3) coalescence (fusion) of more than two droplets. The mechanisms are usually called (1) Siggia's mechanism, (2) Ostwald ripening (or the Lifshitz-Slyozov mechanism), and (3) coalescence.
3. Morphology coarsening can be substantially stopped by, for example, vitrification, crosslinking, and **pinning**, the slowing down of molecular diffusion across domain interfaces.

Cold crystallization: Polymer crystallization brought about from a glass, from a liquid crystalline state, or a state displaying a low degree of crystallinity, at temperatures a little above the glass-transition temperature.

Glass transition: Process in which a polymer melt changes on cooling to a polymer glass or a polymer glass changes on heating to a polymer melt.

1. Phenomena occurring at the glass transition of polymers are still objects of ongoing scientific investigation and debate. The glass transition presents features of a second-order transition since thermal studies often indicate that the molar Gibbs energy, the enthalpy, and the specific volume of the two phases, i.e., the melt and the glass, are equal at the glass-transition temperature, while the heat capacity and the coefficient of thermal expansion are discontinuous. However, the glass transition is generally not regarded as a thermodynamic transition in view of the inherent difficulty in reaching equilibrium both in a polymer glass or in a polymer melt at temperatures close to the glass transition.
2. In the case of polymers, conformational changes of segments, consisting typically of 10-20 main chain atoms, become infinitely slow below the glass-transition temperature.
3. In crystallizable polymers, heating a polymer glass above the glass transition, very frequently leads to some crystallization, allowed by the onset of greater segmental mobility.

Glass-transition temperature (T_g): Temperature at which the glass transition occurs.

1. Crystallization rates of crystallizable polymers below T_g become infinitely slow.
2. The value of T_g measured depends on the cooling or heating rate used, on the analytical technique used in its determination, and, in many instances, on previous thermal treatment.
3. The glass-transition temperature can be determined by a number of different techniques, ranging from thermal analysis to dielectric relaxation, dynamic mechanical analysis, density, viscosity measurements, and spectroscopies which allow the determination of relaxation times.
4. It may often be more useful to refer to a temperature range over which the glass transition occurs, rather than to a single transition temperature.

Supplementary Note 4. Glass transition temperature measurement

Differential scanning calorimetry (DSC) measurements were carried out on a Mettler Toledo DSC 3+ equipped with FRS 6+ sensor and Huber TC 100 intracooler, using Al light 20 µl crucible. A heat-cool-heat cycle with heating/cooling rate of 10 °C/min was applied in the temperature of 0 to 300 °C (except for DR3TSBDT, the temperature range was -50 to 250 °C). To enhance the signal and better locate T_g, a blank curve using empty crucible was performed and later subtracted from the sample heat flow, and T_g was determined using the half-step method. DSC scans of DR3TSBDT, DTS(PTTh$_2$)$_2$, DPPEZnP-TEH, PC$_{61}$BM, PC$_{71}$BM, PBDB-T, PM6 are shown in Supplementary Fig. 13-19. Step change in heat flow, an indicative of glass transition, was observed for those samples. T_g was determined using the half-step method and marked in the figures.

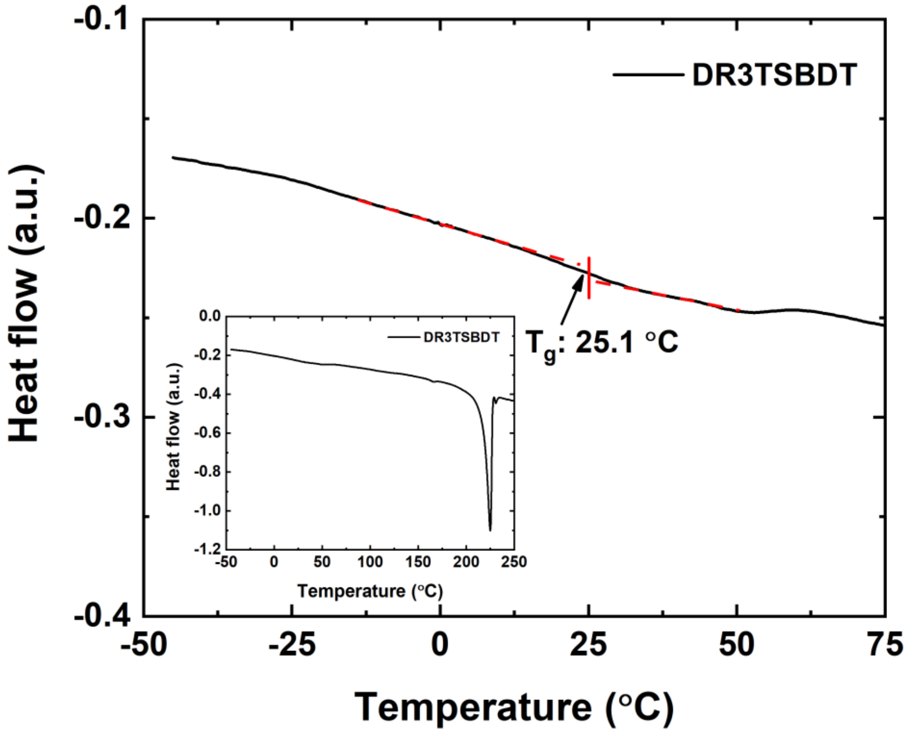

Supplementary Fig. 13 DSC scan of DR3TSBDT. The red dashed lines are glassy line and liquid line. The red vertical line marks the T_g. The inset shows DSC scan in the full temperature range.

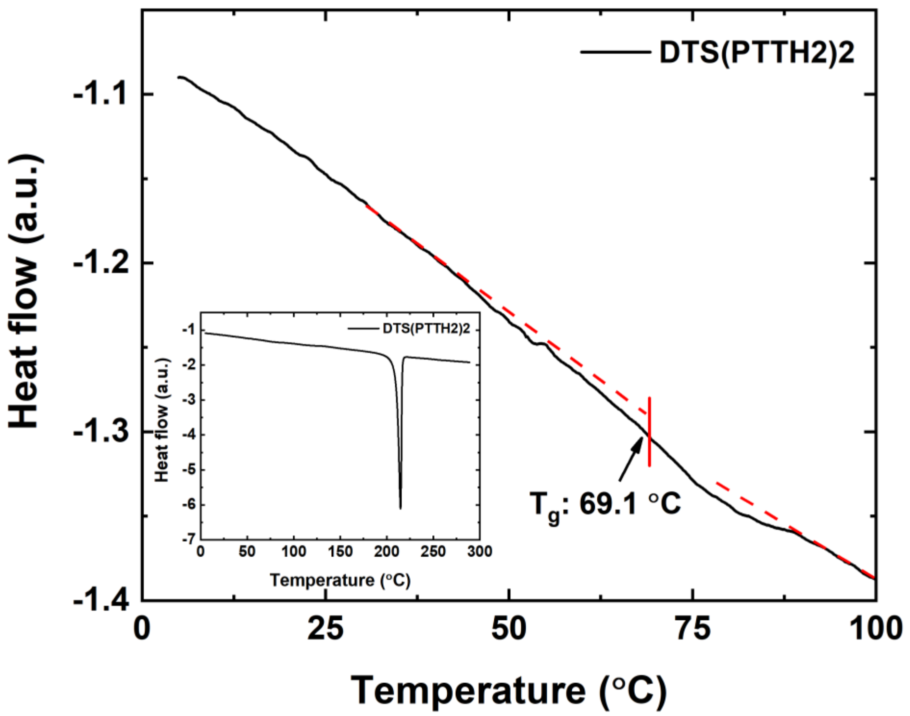

Supplementary Fig. 14 DSC scan of DR3TSBDT. The red dashed lines are glassy line and liquid line. The red vertical line marks the T_g. The inset shows DSC scan in the full temperature range.

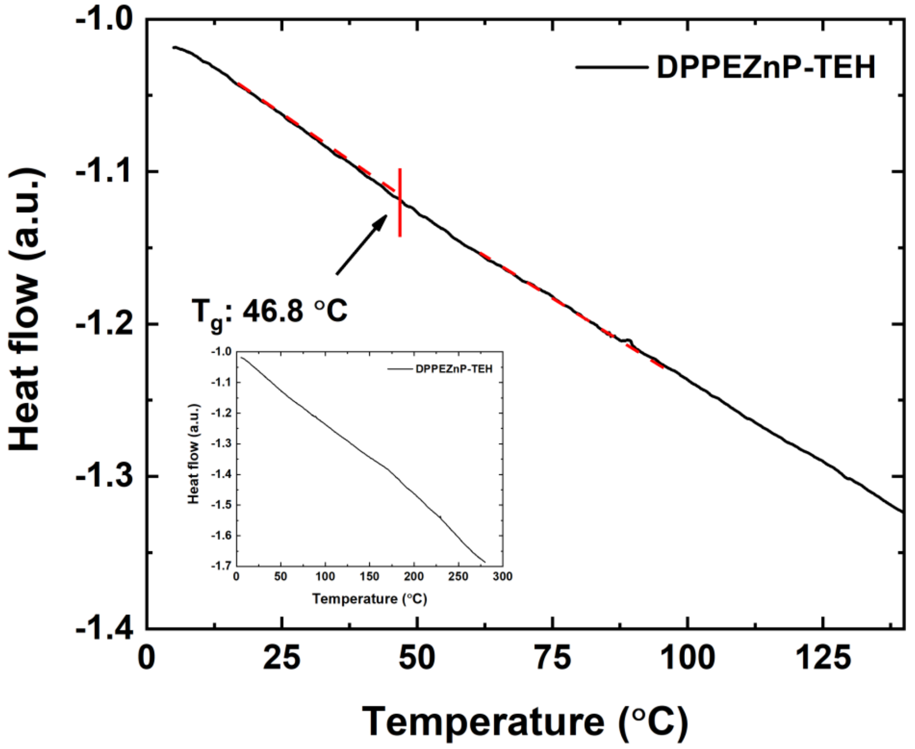

Supplementary Fig. 15 DSC scan of DPPEZnP-TEH. The red dashed lines are glassy line and liquid line. The red vertical line marks the T_g.

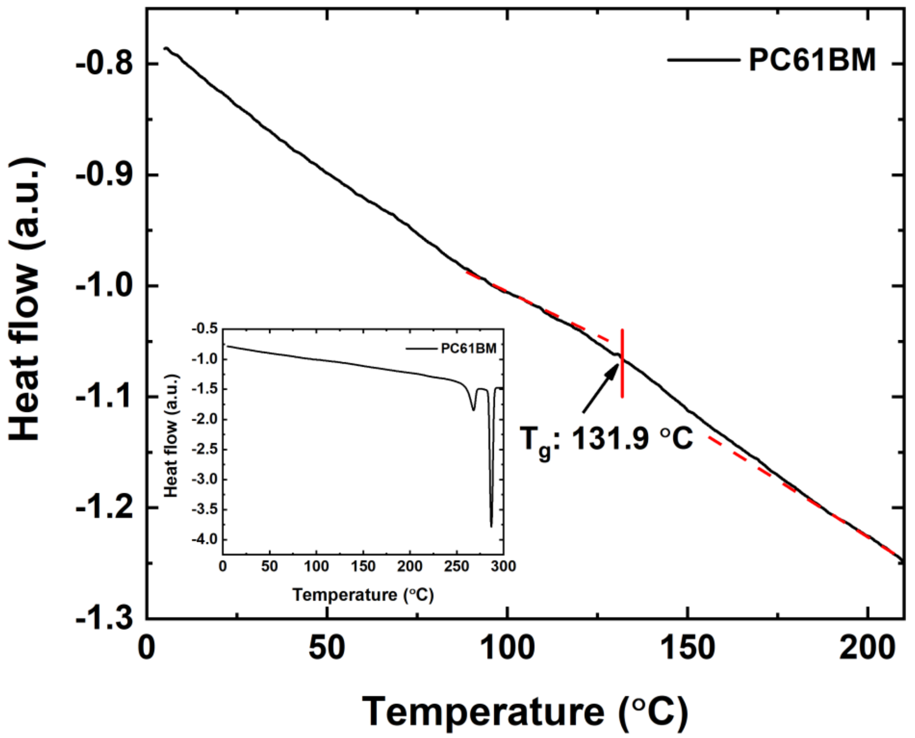

Supplementary Fig. 16 DSC scan of $PC_{61}BM$. The red dashed lines are glassy line and liquid line. The red vertical line marks the T_g. The inset shows DSC scan in the full temperature range.

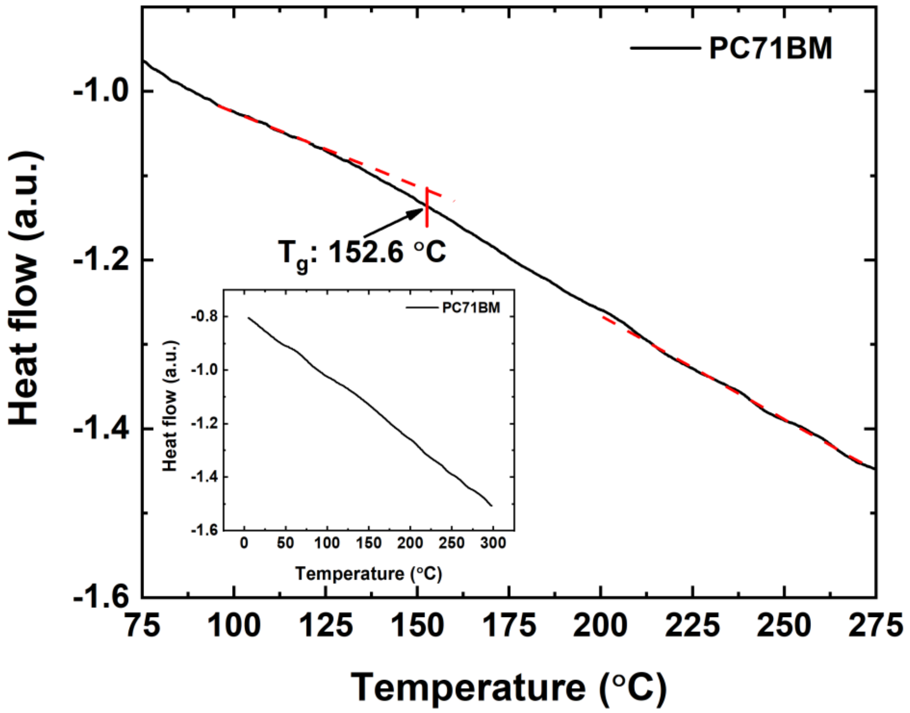

Supplementary Fig. 17 DSC scan of $PC_{71}BM$. The red dashed lines are glassy line and liquid line. The red vertical line marks the T_g. The inset shows DSC scan in the full temperature range.

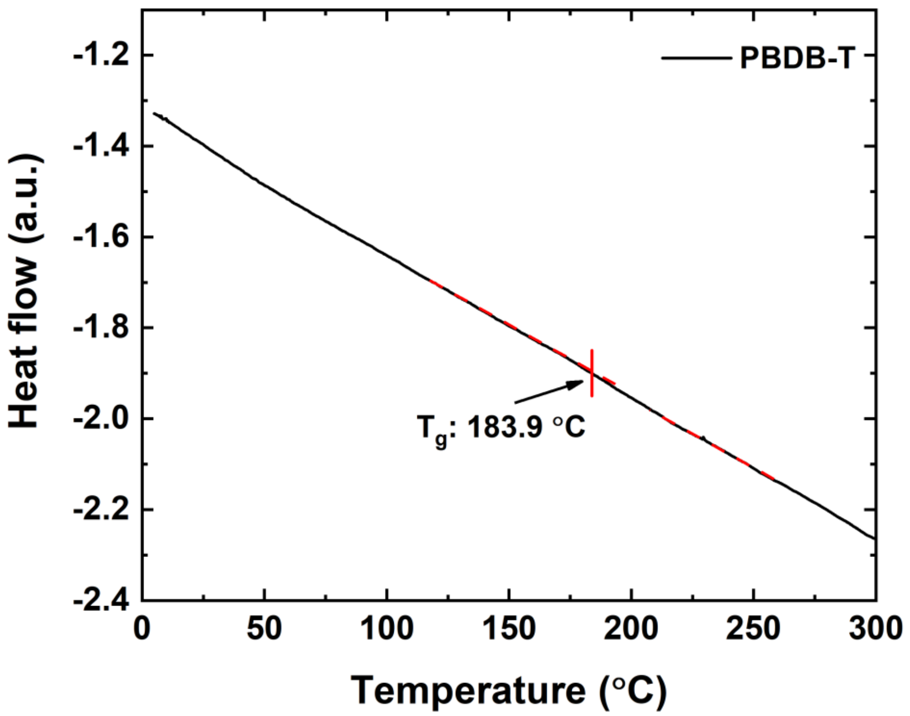

Supplementary Fig. 18 DSC scan of PBDB-T. The red dashed lines are glassy line and liquid line. The red vertical line marks the T_g.

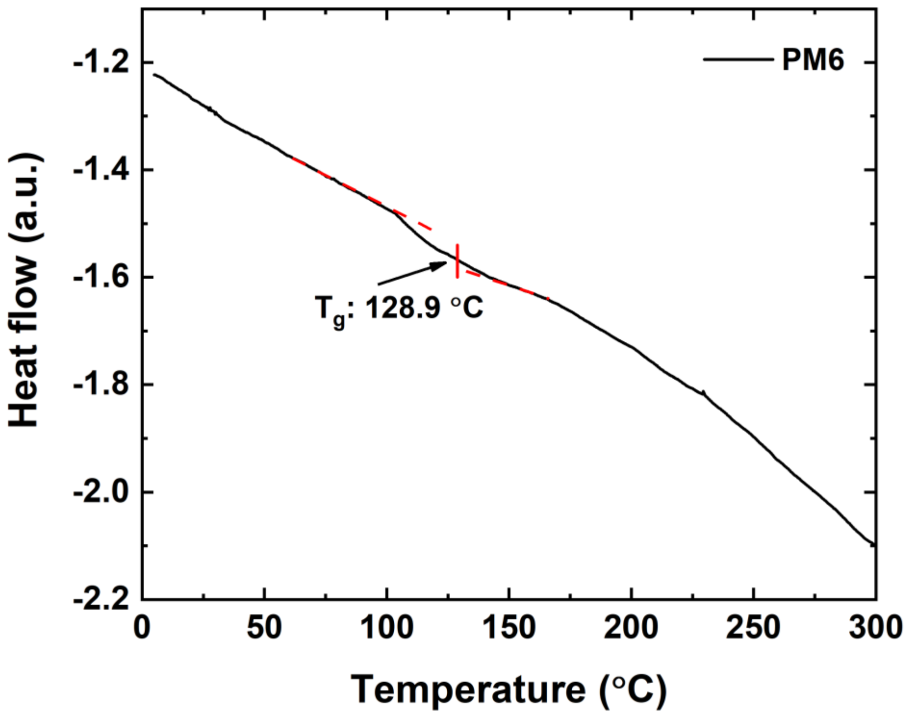

Supplementary Fig. 19 DSC scan of PM6. The red dashed lines are glassy line and liquid line. The red vertical line marks the T_g.

Supplementary Note 5. Melting Point Depression

Melting point depression experiments was carried out using differential scanning calorimetry (DSC) to evaluate interaction parameter χ between high crystalline small molecule and $PC_{71}BM$[26]. The samples were prepared by adding little amount of $PC_{71}BM$ into small molecule (2~25 wt %). These blend samples were dissolved in chloroform and then drop casted to DSC pan. Then use DSC (TA Instruments, Q2000) to check the melting point change. The samples were firstly heated at a rate of 10 °C/min to 260 °C and held for 5 minutes. Subsequently the samples were cooled to 0 °C at 2 °C/min and held for 5 minutes. Then the samples were heated again at a rate of 10 °C/min to 260 °C, and the melting point (T_m) was obtained from the high temperature side intersection of the base line with the tangent to the endotherm. ${T_m}^0$ is the melting point of the pure small molecule obtained using the same procedure.

The interaction parameter χ was determined by fitting the melting point depression data to Supplementary Equation (6):

$$\frac{1}{T_m} - \frac{1}{{T_m}^0} = -\frac{RV_{2u}}{\Delta H_{2u} V_{1u}}\left[\frac{\ln \nu_2}{m_2} + \left(\frac{1}{m_2} - \frac{1}{m_1}\right) \times (1-\nu_2) + \chi_{12}(1-\nu_2)^2\right] \quad (6)$$

where ${T_m}^0$ represents the melting point of host matter in the standard state, T_m is the melting point when mixed with impurities, R is the gas constant, the subscript 1 identified with the impurities and 2 with the host matter, V_u is the molar volume (of repeating unit for polymer), ΔH_u is enthalpy of fusion per mole (of repeating unit for polymer), ν is the volume fraction, m is the degree of polymerization, and χ_{12} represents the host-impurities interaction parameter.

In our case, the host matter is small molecule and $PC_{71}BM$ can be regarded as impurities. Both m_1 and m_2 were taken as 1, and the density value of 1.1 g/cm^3 and 1.4 g/cm^3 were used for small molecule and $PC_{71}BM$ in fitting process.

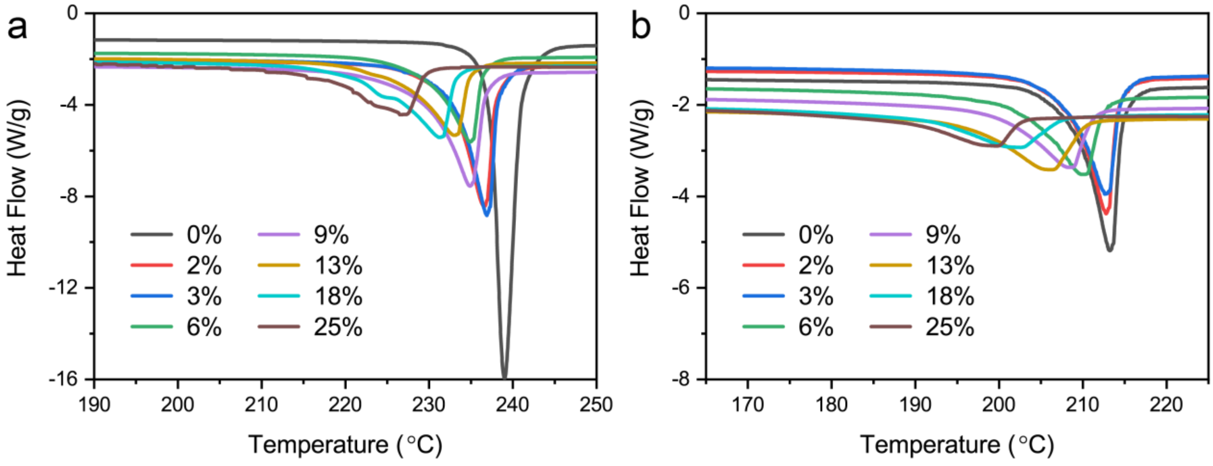

Supplementary Fig. 20 Differential scanning calorimetry (DSC) endothermal curves of (**a**) DR3TSBDT and (**b**) DTS(PTTh$_2$)$_2$ with different mass ratio of PC$_{71}$BM to show melting point depression behavior.

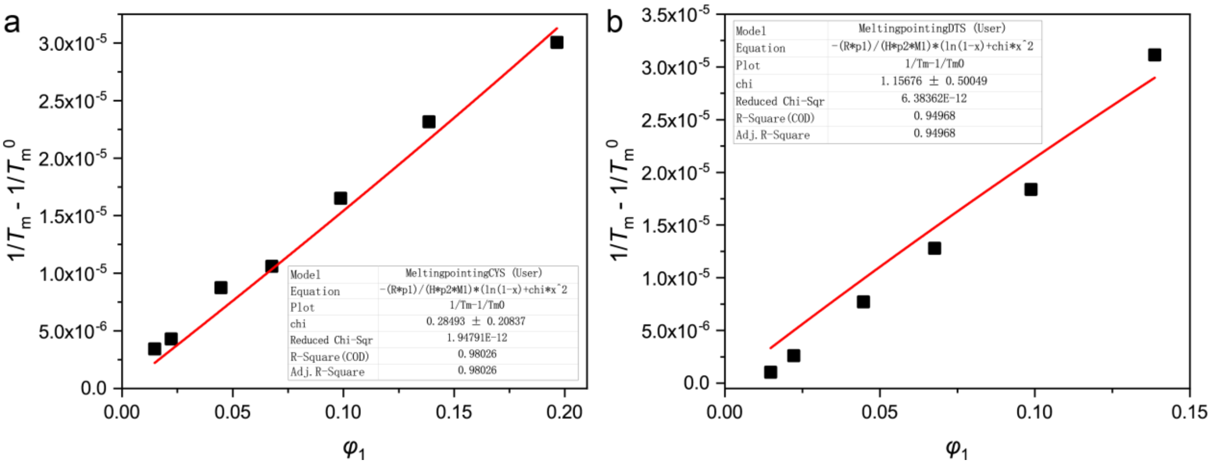

Supplementary Fig. 21 Interaction parameter χ fitting results of (**a**) DR3TSBDT and (**b**) DTS(PTTh$_2$)$_2$. (φ_1 is the volume fraction of PC$_{71}$BM).

Supplementary Note 6. Device fabrication and performance characterization

The devices were fabricated on pre-patterned ITO glass substrates using an ITO/PEDOT:PSS/SM:fullerene/Al structure, except for DTS(PTTh$_2$)$_2$:PC$_{71}$BM using a ITO/PFN/SM:fullerene/MoO$_x$/Al structure. After ultrasonically cleaned in detergent solution, deionized water, acetone and isopropyl alcohol, the substrates were dried in nitrogen flow and treated by UV-Ozone for 15 min. A 30 nm PEDOT:PSS (Clevious P VP AI 4083 H. C. Stark, Germany) or 10 nm thick PFN was then spin-coated onto the ITO substrates. The substrate with PEDOT:PSS were baked at 150 °C for 15 min. The substrates were then transferred into nitrogen glove box. All the active films were spin-coated following the processing conditions shown in earlier reports (shown in Supplementary Table 2)[1-3]. Annealing were applied subsequently. A 10 nm MoO$_x$ layer (for DTS(PTTh$_2$)$_2$: PC$_{71}$BM only) and a 100 nm Al layer were sequentially thermally evaporated through a shadow mask to define the active area of the devices (3.14 mm^2) and form the top cathode. All device fabrication processes are carried out in a nitrogen glove box at room temperature. The PCE was determined from *J-V* curve measurements (using a Keithley 2400 Source Meter) under a 100 mW cm^{-2}, AM 1.5G spectrum from a solar simulator (Oriel model 91192). Masks made using laser beam cutting technology to have a well-defined area of 3.14 mm^2 were attached to define the effective area for accurate measurement. The solar simulator illumination intensity was determined using a monocrystal silicon reference cell (Hamamatsu S1133, with KG-5 visible color filter) calibrated by the National Renewable Energy Laboratory (NREL). The *J-V* curves were measured along the forward scan direction from -0.2 to 1.5 V or the reverse scan direction from 1.5 to -0.2 V, yielding identical results. The scan speed and dwell times were fixed at 0.015 V s^{-1} and 20 ms, respectively.

Supplementary Table 2. Summary of device performance DR3TSBDT:PC$_{71}$BM, DTS(PTTh$_2$)$_2$:PC$_{71}$BM, DPPEZnP-TEH:PC$_{61}$BM and DPPEZnP-TEH:PC$_{61}$BM/Py with different annealing time

Active layer	t_A (min)	V_{OC} (V)	J_{SC} (mA/cm^2)	*FF* (%)	PCE (%)
DR3TSBDT:PC$_{71}$BM (1:0.8 w/w, 14.4 mg/ml, CF), 1700 rpm 100 °C TA	0	0.991±0.005	11.87±0.54	56.1±1.0	6.57±0.24
	0.5	0.990±0.005	12.01±0.78	55.4±1.3	6.59±0.52
	1	0.989±0.005	12.07±0.66	56.9±1.1	6.79±0.35
	5	0.984±0.003	12.32±0.83	56.7±2.7	6.89±0.67
	10	0.979±0.005	12.85±0.66	59.4±0.5	7.52±0.45
	30	0.976±0.003	13.12±0.89	61.4±0.9	7.89±0.60
DTS(PTTh$_2$)$_2$:PC$_{71}$BM (1:2.3 w/w, 40 mg/ml, CB), 1500 rpm 110 °C TA	0	0.856±0.005	4.43±0.25	28.6±0.8	1.06±0.15
	0.5	0.850±0.005	8.19±0.31	38.2±0.4	2.67±0.09
	1	0.825±0.015	10.01±0.70	44.4±1.7	3.65±0.23
	5	0.823±0.006	11.04±0.82	46.2±1.0	4.12±0.33
	10	0.815±0.005	10.14±0.44	49.4±0.9	4.07±0.14
	30	0.806±0.004	9.97±0.74	49.1±0.6	3.96±0.27
DPPEZnP-TEH:PC$_{61}$BM	0	0.794±0.005	9.42±0.52	59.6±1.1	4.47±0.34

(1:1.2 w/w, 39.6 mg/ml,	0.5	0.793±0.004	9.58±0.81	60.9±0.5	4.63±0.42
CB), 1450 rpm	1	0.789±0.007	9.80±0.44	58.8±3.5	4.54±0.38
110 °C TA	5	0.794±0.005	10.02±0.38	60.5±0.3	4.81±0.22
	10	0.793±0.005	9.91±0.37	58.7±3.8	4.61±0.37
	30	0.790±0.002	9.83±0.75	55.9±0.7	4.34±0.31
	0	0.827±0.004	9.23±0.35	31.9±0.8	2.42±0.22
DPPEZnP-TEH:$PC_{61}BM$/Py	0.5	0.825±0.005	16.82±0.26	47.6±1.1	6.61±0.18
(1:1.2 w/w, 39.6 mg/ml, CB	1	0.822±0.004	16.84±0.27	48.5±0.5	6.71±0.14
+ 1% pyridine), 1450 rpm	5	0.811±0.003	17.96±0.50	50.5±0.4	7.36±0.26
110 °C TA	10	0.806±0.005	17.62±0.51	51.3±0.7	7.29±0.25
	30	0.796±0.005	17.66±0.23	50.5±1.2	7.11±0.24

For PBDB-T:ITIC, devices were fabricated using an ITO/PEDOT:PSS/PBDB-T:ITIC/ PDINO/Al structure according to the following procedure. Patterned ITO glass substrates were sequential cleaned by ultrasonicating in acetone, detergent, deionized water and isopropyl alcohol for 15 min each and then dried under dry oven. The precleaned substrates were treated in an ultraviolet-ozone chamber for 15 min, then a ~40 nm thick PEDOT:PSS (Clevious P VP AI 4083 H. C. Stark, Germany) thin film was deposited onto the ITO surface by spin-coating and baked at 150 °C for 15 min. D:A ratio was 1:1 and dissolved in chlorobenzene solution at a polymer concentration of 10 mg/ml (1% DIO was added as solvent additive). The active layers were spin-coated to a film thickness of 100 nm. The prepared films were treated with different thermal annealing temperature for 10 min. After cooling to room temperature, a ~5 nm thick of PNDIT-F3N-Br was spin-coated on the top of active layer. Then, those samples were brought into to an evaporate chamber and a 150 nm thick silver layer was thermally evaporated on the PNDIT-F3N-Br layer at a base pressure of 1×10^{-6} mbar. The evaporation thickness was controlled by SQC-310C deposition controller (INFICON, Germany). Twelve devices were fabricated on one substrate and the active area of each device was 0.05 cm^2 defined by a shadow mask.

For PCE10:IEICO-4F, devices were fabricated using an ITO/PEDOT:PSS/PCE10:IEICO-4F /PNDIT-F3N-Br/Ag structure. D:A ratio was 1:1.5 and dissolved in chlorobenzene solution at a polymer concentration of 10 mg/ml (1% DIO was added as solvent additive). The active layers were spin-coated to a film thickness of 100 nm.

For PM6:Y6, devices were fabricated using an ITO/PEDOT:PSS/PM6:Y6/PNDIT-F3N-Br/ Ag structure. D:A ratio was 1:1.2 and dissolved in chlorobenzene solution at a total concentration of 14 mg/ml with 0.5% 1-chloronaphthalene (CN) as additive. The active layers were spin-coated to a film thickness of 140 nm with a speed of 2300 rpm.

For PM6:ITIC-4F, devices were fabricated using an ITO/PEDOT:PSS/PM6:ITIC-4F/PNINO/ Al structure. D:A ratio was 1:1 and dissolved in chlorobenzene solution at a total concentration of 20 mg/ml (0.75% volume of DIO was added as solvent additive).

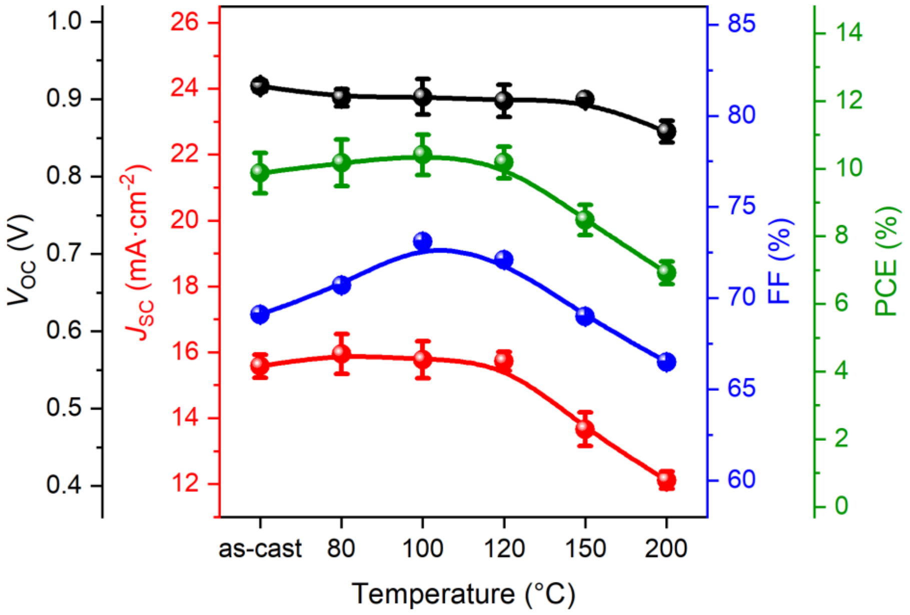

Supplementary Fig. 22 PBDB-T:ITIC device performance of V_{OC}, J_{SC}, FF and PCE.

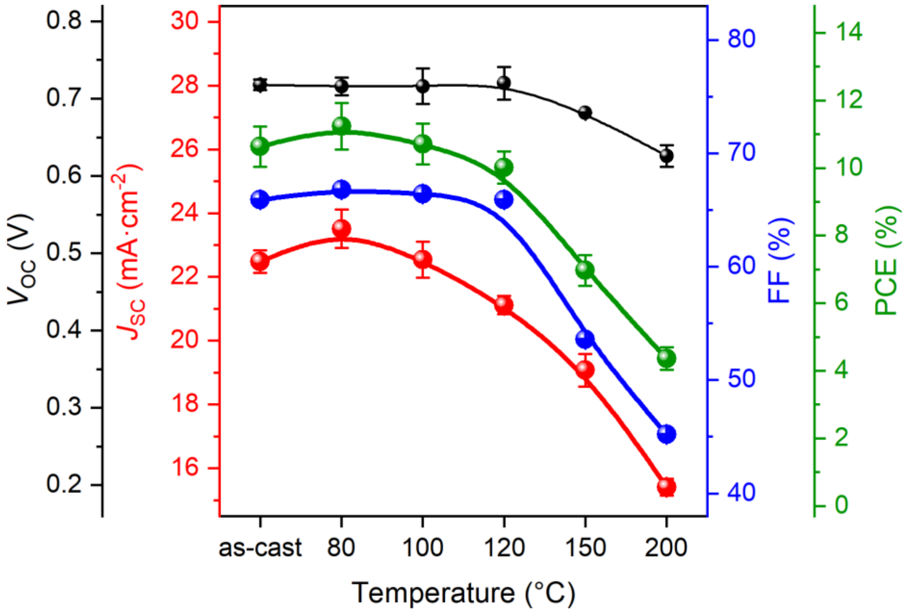

Supplementary Fig. 23 PCE-10:IEICO-4F device performance of V_{OC}, J_{SC}, FF and PCE.

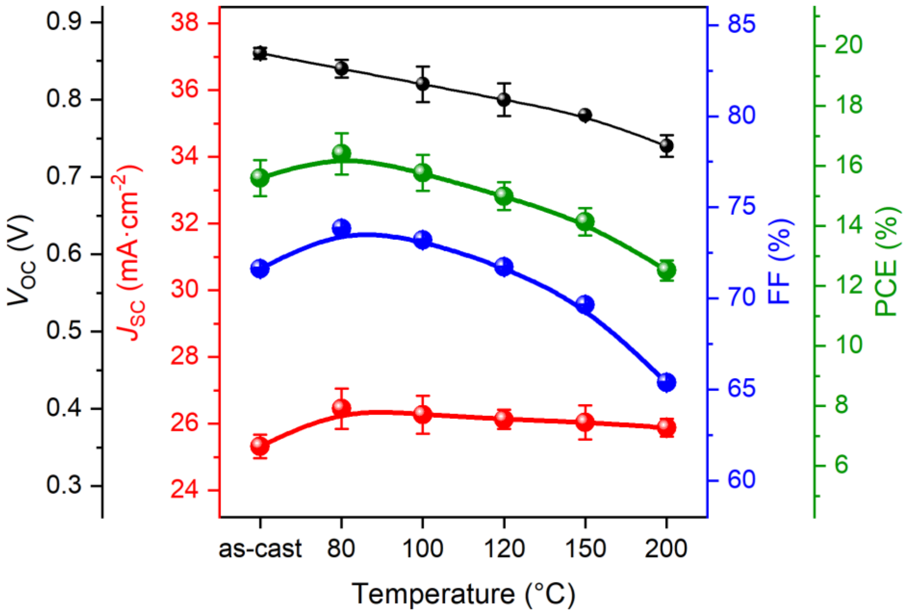

Supplementary Fig. 24 PM6:Y6 device performance of V_{OC}, J_{SC}, FF and PCE.

Supplementary References

1. Kan, B., *et al.* Solution-processed organic solar cells based on dialkylthiol-substituted benzodithiophene unit with efficiency near 10%. *J. Am. Chem. Soc.* **136**, 15529-15532 (2014).
2. Sun, Y., Welch, G. C., Leong, W. L., Takacs, C. J., Bazan, G. C. & Heeger, A. J. Solution-processed small-molecule solar cells with 6.7% efficiency. *Nat. Mater.* **11**, 44-48 (2012).
3. Gao, K., *et al.* Deep absorbing porphyrin small molecule for high-performance organic solar cells with very low energy losses. *J. Am. Chem. Soc.* **137**, 7282-7285 (2015).
4. Qian, D., *et al.* Design, application, and morphology study of a new photovoltaic polymer with strong aggregation in solution state. *Macromolecules* **45**, 9611–9617 (2012)
5. Zhang, M., Guo, X., Ma, W., Ade, H. & Hou, J. A large-bandgap conjugated polymer for versatile photovoltaic applications with high performance. *Adv. Mater.* **27**, 4655-4660 (2015)
6. Zhang, S., Ye, L., Zhao, W., Liu, D., Yao, H. & Hou, J. Side chain selection for designing highly efficient photovoltaic polymers with 2D-conjugated structure. *Macromolecules* **47**, 4653–4659 (2014)
7. Lin, Y., Wang, J., Zhang, Z.-G., Bai, H., Li, Y., Zhu, D. & Zhan, X. An electron acceptor challenging fullerenes for efficient polymer solar cells. *Adv. Mater.* **27**, 1170-1174 (2015)
8. Zhao, W., Li, S., Yao, H., Zhang, S., Zhang, Y., Yang, B. & Hou, J. Molecular optimization enables over 13% efficiency in organic solar cells. *J. Am. Chem. Soc.* **139**, 7148–7151 (2017)
9. Yao, H., *et al.* Design and synthesis of a low bandgap small molecule acceptor for efficient polymer solar cells. *Adv. Mater.* **28**, 8283–8287 (2016)
10. Yuan, J., et al. Single-junction organic solar cell with over 15% efficiency using fused-ring acceptor with electron-deficient core. *Joule* **3**, 1140-1151 (2019)
11. Hexemer, A., *et al.* A SAXS/WAXS/GISAXS beamline with multilayer monochromator. In: *14th International Conference on Small-Angle Scattering (SAS09)* (ed^(eds). Iop Publishing Ltd (2009).
12. Ilavsky, J. Nika: software for two-dimensional data reduction. *J. Appl. Cryst.* **45**, 324-328 (2012)
13. Gann, E., *et al.* Soft X-ray scattering facility at the advanced light source with real-time data processing and analysis. *Rev. Sci. Instrum.* **83**, 045110 (2012).
14. Smilgies, D. M. Scherrer grain-size analysis adapted to grazing-incidence scattering with area detectors. *J. Appl. Crystallogr.* **42**, 1030-1034 (2009).
15. Scherrer, P. Determination of size and internal structure of colloid particles by X-rays. *Nachr Ges wiss goettingen Math. Phys.* **2**, 98-100 (1918).
16. Noriega, R., *et al.* A general relationship between disorder, aggregation and charge transport in conjugated polymers. *Nat. Mater.* **12**, 1038–1044 (2013).
17. Rivnay, J., Noriega, R., Kline, R. J., Salleo, A., & Toney, M. F. Quantitative analysis of lattice disorder and crystallite size in organic semiconductor thin films. *Phys. Rev. B* **84**, 045203 (2011).
18. Rivnay, J., Mannsfeld, S. C. B., Miller, C. E., Salleo, A. & Toney M. F. Quantitative determination of organic semiconductor microstructure from the molecular to device scale. *Chem. Rev.* **112**, 5488−5519 (2012)
19. Savikhin, V., *et al.* Impact of polymer side chain modification on OPV morphology and performance. *Chem. Mater.* **30**, 7872−7884 (2018)
20. Hammouda, B., Ho, D. L. & Kline, S. Insight into clustering in poly(ethylene oxide) solutions. *Macromolecules* **37**, 6932-6937 (2004).

21. Aleman, J., *et al.* Definitions of terms relating to the structure and processing of sols, gels, networks, and inorganic-organic hybrid materials (IUPAC recommendations 2007). *Pure Appl. Chem.* **79**, 1801-1827 (2007).
22. Work, W. J., Horie, K., Hess, M. & Stepto, R. F. T. Definitions of terms related to polymer blends, composites, and multiphase polymeric materials (IUPAC recommendations 2004). *Pure Appl. Chem.* **76**, 1985-2007 (2004).
23. Jones, R. G., Ober, C. K., Hodge, P., Kratochvil, P., Moad, G. & Vert, M. Terminology for aggregation and self-assembly in polymer science (IUPAC recommendations 2013). *Pure Appl. Chem.* **85**, 463-490 (2013).
24. Meille, S. V., *et al.* Definitions of terms relating to crystalline polymers (IUPAC recommendations 2011). *Pure Appl. Chem.* **83**, 1831-1871 (2011).
25. Baron, M. Definitions of basic terms relating to low-molar-mass and polymer liquid crystals (IUPAC recommendations 2001). *Pure Appl. Chem.* **73**, 845-895 (2001).
26. Liu, F., *et al.* Molecular weight dependence of the morphology in P3HT:PCBM solar cells. *ACS Appl. Mater. Interfaces* **6**, 19876-19887 (2014).
