# Supplementary material for: The coupling and competition of crystallization and phase separation, correlating thermodynamics and kinetics in OPV morphology and performances
Source: Nat Commun. 2021 Jan 12;12:332. doi: 10.1038/s41467-020-20515-3 (PMC7804468; doi:10.1038/s41467-020-20515-3)
Supplement: Supplementary file 3 — Solar Cells Reporting Summary [file 41467_2020_20515_MOESM3_ESM.pdf]

nature research

Corresponding author(s): Ke Gao, Alex K.-Y. Jen and Feng Liu

Solar Cells Reporting Summary

Nature Research wishes to improve the reproducibility of the work that we publish. This form is intended for publication with all accepted papers reporting the characterization of photovoltaic devices and provides structure for consistency and transparency in reporting. Some list items might not apply to an individual manuscript, but all fields must be completed for clarity.

For further information on Nature Research policies, including our data availability policy, see Authors & Referees.

Experimental design

Please check: are the following details reported in the manuscript?

1. Dimensions

Item	Reported	Details
Area of the tested solar cells	[x] Yes [ ] No	See "Device fabrication and characterization" in Supplementary Information.
Method used to determine the device area	[x] Yes [ ] No	The area of our device was certified at National Institute of Metrology, China (NIM).

2. Current-voltage characterization

Item	Reported	Details
Current density-voltage (J-V) plots in both forward and backward direction	[x] Yes [ ] No	Both forward and backward scans were conducted, which yielded identical results.
Voltage scan conditions<br>*For instance: scan direction, speed, dwell times*	[x] Yes [ ] No	See "Device fabrication and characterization" in Supplementary Information.
Test environment<br>*For instance: characterization temperature, in air or in glove box*	[x] Yes [ ] No	See "Device fabrication and characterization" in Supplementary Information.
Protocol for preconditioning of the device before its characterization	[ ] Yes [x] No	No preconditioning protocol.
Stability of the J-V characteristic<br>*Verified with time evolution of the maximum power point or with the photocurrent at maximum power point; see ref. 7 for details.*	[x] Yes [ ] No	The stability were obtained by tracking the maximum output power point.

3. Hysteresis or any other unusual behaviour

Item	Reported	Details
Description of the unusual behaviour observed during the characterization	[ ] Yes [x] No	No hysteresis or other unusual behaviour was observed during the characterization of the solar cells. In general, organic solar cells do not have hysteresis problems.
Related experimental data	[ ] Yes [x] No	No hysteresis or other unusual behaviour was observed during the characterization of the solar cells.

4. Efficiency

Item	Reported	Details
External quantum efficiency (EQE) or incident photons to current efficiency (IPCE)	[x] Yes [ ] No	The EQE measurements were conducted. The integrated response under the standard reference spectrum are quite comparable to the response measured under the simulator with a error less than 5%.
A comparison between the integrated response under the standard reference spectrum and the response measure under the simulator	[x] Yes [ ] No	The EQE measurements were conducted. The integrated response under the standard reference spectrum are quite comparable to the response measured under the simulator with a error less than 5%.
For tandem solar cells, the bias illumination and bias voltage used for each subcell	[ ] Yes [x] No	Devices are single solar cells structure only.

5. Calibration

Item	Reported	Details
Light source and reference cell or sensor used for the characterization	[x] Yes [ ] No	See "Device fabrication and characterization" in Supplementary Information.
Confirmation that the reference cell was calibrated and certified	[x] Yes [ ] No	See "Device fabrication and characterization" in Supplementary Information.

November 2017

Calculation of spectral mismatch between the reference cell and the devices under test	☒ Yes ☐ No	The spectral mismatch factor has been included in testing.

6. Mask/aperture

Size of the mask/aperture used during testing	☒ Yes ☐ No	See "Device fabrication and characterization" in Supplementary Information.
Variation of the measured short-circuit current density with the mask/aperture area	☐ Yes ☒ No	Variation is small.

7. Performance certification

Identity of the independent certification laboratory that confirmed the photovoltaic performance	☐ Yes ☒ No	We did not confirm the photovoltaic performance from independent certification laboratories, as our aim is not to develop new devices or break efficiency record.
A copy of any certificate(s) *Provide in Supplementary Information*	☐ Yes ☒ No	No copy is provided as we did not confirm the photovoltaic performance from independent certification laboratories.

8. Statistics

Number of solar cells tested	☒ Yes ☐ No	For each condition, 10 devices were tested for statistics.
Statistical analysis of the device performance	☒ Yes ☐ No	The statistical analysis can be found in error bars of Fig. 8 and average performance data in Supplementary Table 2.

9. Long-term stability analysis

Type of analysis, bias conditions and environmental conditions *For instance: illumination type, temperature, atmosphere humidity, encapsulation method, preconditioning temperature*	☐ Yes ☒ No	As our current focus is on morphology evolution of organic solar cells based on reported systems, we did not carry out systematic study on long-term stability track.
